# Sources of PM_2.5_‐Associated Health Risks in Europe and Corresponding Emission‐Induced Changes During 2005–2015

**DOI:** 10.1029/2022GH000767

**Published:** 2023-03-20

**Authors:** Yixuan Gu, Daven K. Henze, M. Omar Nawaz, Hansen Cao, Ulrich J. Wagner

**Affiliations:** ^1^ Department of Mechanical Engineering University of Colorado Boulder Boulder CO USA; ^2^ Department of Economics University of Mannheim Mannheim Germany; ^3^ Department of Chemistry University of York York UK

**Keywords:** source appointment, PM_2.5_, health impacts, adjoint, Europe

## Abstract

We present a newly developed approach to characterize the sources of fine particulate matter (PM_2.5_)‐related premature deaths in Europe using the chemical transport model GEOS‐Chem and its adjoint. The contributions of emissions from each individual country, species, and sector are quantified and mapped out at km scale. In 2015, total PM_2.5_‐related premature death is estimated to be 449,813 (257,846–722,138) in Europe, 59.0% of which were contributed by domestic anthropogenic emissions. The anthropogenic emissions of nitrogen oxides, ammonia, and organic carbon contributed most to the PM_2.5_‐related health damages, making up 29.6%, 23.2%, and 16.8%, respectively of all domestic anthropogenic contributions. Residential, agricultural, and ground transport emissions are calculated to be the largest three sectoral sources of PM_2.5_‐related health risks, accounting for 23.5%, 23.0%, and 19.4%, respectively, of total anthropogenic contributions within Europe. After excluding the influence of extra‐regional sources, we find eastern European countries suffered from more premature deaths than their emissions caused; in contrast, the emissions from some central and western European regions contributed premature deaths exceeding three times the number of deaths that occurred locally. During 2005–2015, the first decade of PM_2.5_ regulation in Europe, emission controls reduced PM_2.5_‐related health damages in nearly all European countries, resulting in 63,538 (46,092–91,082) fewer PM_2.5_‐related premature deaths. However, our calculation suggests that efforts to reduce air pollution from key sectors in some countries can be offset by the lag in control of emissions in others. International cooperation is therefore vitally important for tackling air pollution and reducing corresponding detrimental effects on public health.

## Key Points

- Residential, agricultural, and ground transport emissions were the largest sources of the PM_2.5_‐related health burden in Europe
- Eastern Europe experienced more premature deaths than their own emissions caused, making them net importers of the pollution health burden
- Emission reductions, most from transport, energy, and industrial sources, reduced pollution health damages in Europe during 2005–2015

## 1 Introduction

Outdoor air pollution has been a top global health concern since the 1970s (Crippa et al., 2016; Fenger, 2009; McKitrick, 2007), and has been a leading cause of the global disease burden for decades (Burnett et al., 2018; Cohen et al., 2005; C. J. L. Murray et al., 2020). Long‐term exposure to outdoor pollution, mostly by exposure to fine particulate matter (with an aerodynamic diameter smaller than 2.5 μm; PM_2.5_), was calculated to lead to 3.3 million premature deaths worldwide in 2010 (Lelieveld et al., 2015), and quantifications of ambient PM_2.5_‐related health risks implied an annualized growth rate of 1.46% during 2010–2019 (C. J. L. Murray et al., 2020). Epidemiologic cohort studies have provided increasing evidence that PM_2.5_ exposure increases the risk of premature death from health outcomes including chronic obstructive pulmonary disorder (COPD), ischemic heart disease (IHD), lower respiratory illnesses (LRI), lung cancer (LC), type‐II diabetes (T2D), and stroke (Anderson et al., 2012; Pinault et al., 2017; Thurston et al., 2016; Yin et al., 2017). Owing to this, developing effective strategies for reducing the burden of disease attributable to PM_2.5_ exposure is a sustainability goal shared by countries worldwide.

During the past two decades, the European Union (EU) has enacted multiple policies to reduce air pollution. In 2005, a cap of 25 μg m^−3^ for the annual average exposure to PM_2.5_ was first proposed to reduce the exposure of the population in addition to the existing controls on PM_10_ (with aerodynamic diameter less than 10 μm), and a uniform reduction target of 20% was proposed for all member states to be attained between 2010 and 2020 (COM (2005) 0446 final, 2005). After that, PM_2.5_ exposure reduction targets were set at the national level by a series of directives, aiming to reduce the annual mean PM_2.5_ concentrations to 25 μg m^−3^ in 2015 and 20 μg m^−3^ in 2020 (Directive, 2008/50/EC, 2008). Emission caps were set for PM_2.5_ and its precursors, like sulfur dioxide (SO_2_), nitrogen oxides (NO_*x*_), ammonia (NH_3_), and non‐methane volatile organic species, in each member state (Directive, 2001/81/EC, 2001). As a result, emissions of PM_2.5_, SO_2_, NO_*x*_, and NH_3_ fell by 29%, 76%, 36%, and 8%, respectively in 2019 compared to those in 2005 in 27 member states of the EU (EU‐27) (EEA, 2021). The World Health Organization (WHO) established a new air quality guideline (AQG) level of 5 μg m^−3^ for long‐term PM_2.5_ exposure in 2021 (WHO, 2021). This new guideline represents the lowest exposure level of PM_2.5_ above which there could be an increase in adverse health impacts. Even in 2020, when anthropogenic emissions were reduced due to the coronavirus pandemic, over 96% of the EU urban population was still exposed to PM_2.5_ concentrations exceeding the AQG level (EEA, 2022). In line with this, Tarín‐Carrasco et al. (2022) estimated that PM_2.5_ accounted for 725,000–1,056,000 annual excess premature deaths across Europe in 2010, and predicted that the number of PM_2.5_‐related deaths would keep increasing in the next 50 years.

To reduce the deleterious impacts of PM_2.5_ pollution on public health, there is a continued need to evaluate the sources of PM_2.5_‐related health impacts in Europe. Chemical transport models (CTMs) are frequently used for such studies due to their unique capabilities of simulating non‐linear processes of atmospheric chemistry, unlike other source apportionment approaches that are limited to primarily linear relationships (Thunis et al., 2019). The effects of emission changes on PM_2.5_‐associated health impacts have previously been characterized by comparing simulated results under different emission scenarios (Andersson et al., 2009; Anenberg et al., 2014; Crippa et al., 2019; Im et al., 2018; Lelieveld et al., 2015, 2019; Silva, Adelman, et al., 2016; Silva, West, et al., 2016; Tarín‐Carrasco et al., 2022). For example, Lelieveld et al. (2015) quantified the contributions of seven source categories to PM_2.5_‐related premature deaths in 2010 by removing their emissions one at a time from ECHAM5/MESSy atmospheric chemistry (EMAC) model simulations and found that agricultural sources could be a leading source category in Europe. The multi‐model results of Im et al. (2018) suggested that a 20% reduction of European anthropogenic emissions could avoid a total of 47,000 premature deaths in Europe. In addition to these brute‐force finite difference calculations, CTM tagging approaches, where emissions of certain species are marked (“tagged”) in the computation so that they can be tracked from particular source categories or locations, are also capable of doing source apportionment. Incorporating the tagging method into an integrated model system, Economic Valuation of Air pollution, Brandt et al. (2013) estimated that emissions from power plant, agricultural, road transport, and non‐industrial combustion plant sources contributed 24%, 25%, 18%, and 10%, respectively of the total health‐related external costs in Europe in 2000. Both these approaches provide valuable insights, but they can be computationally expensive when fine temporal or spatial detail is needed regarding sources, and hence are frequently limited in terms of the resolution of sources that can be considered (Henze et al., 2007, 2009).

Adjoint models provide an alternative approach to efficiently calculate the response of a particular receptor function (e.g., PM_2.5_ concentration, PM_2.5_‐related premature deaths) to a large number of sources, with which detailed contributions from all kinds of emissions can be mapped out in health assessment studies (Lee et al., 2015; Malley et al., 2021; Nawaz & Henze, 2020; Nawaz et al., 2021; Pappin & Hakami, 2013). Compared to traditional model calculations of changes in the final state (e.g., concentrations) induced by a perturbation in model parameters (e.g., emissions), the adjoint method is a receptor‐oriented approach which calculates the sensitivities of the final state to a series of model parameters by transforming the changes in the final state backward in time (Henze et al., 2007, 2009). Based on the adjoint sensitivity calculation, Lee et al. (2015) examined the response of global PM_2.5_‐related mortality to changes in different local emissions in 2005, suggesting that 1 kg km^−2^ yr^−1^ decrease in NH_3_ and carbonaceous aerosol emissions could lead to the largest reductions in global mortality. For the European region, air pollution sensitivity studies and data assimilation have been conducted for over two decades using regional adjoint modeling but without a focus on health impacts (e.g., Elbern & Schmidt, 2001; Elbern et al., 2000; Menut et al., 2000; Vautard et al., 2000). Previous adjoint‐based health impact source attribution studies with respect to this region have been conducted as part of global simulations with a horizontal resolution of 2° × 2.5° in Lee et al. (2015) and Malley et al. (2021). The coarse spatial distributions contribute to significant uncertainties in the health assessments, especially in polluted or populated areas (Li et al., 2016; Punger & West, 2013). Li et al. (2016) quantitatively examined the influence of model resolution on estimates of PM_2.5_‐related premature mortality, suggesting that the calculated national mortality from a coarse‐resolution (2° × 2.5°) simulation could be 8% lower than that from the fine‐resolution (0.5° × 0.666°) model simulation in the United States (US); uncertainty in ascribing this health burden to specific sources of PM_2.5_ precursor emissions would be even higher. From this point of view, approaches to reduce the uncertainty of adjoint simulations are needed to obtain details of the sources of pollution‐associated mortality in Europe.

Here we present a high‐resolution adjoint calculation in Europe by conducting a nested‐grid simulation using the CTM GEOS‐Chem and its adjoint to examine the response of total PM_2.5_‐related premature deaths to various emissions. Remote sensing derived surface‐level PM_2.5_ concentrations are incorporated into the adjoint sensitivity analyses to correct for model biases and to characterize km‐scale spatial variability. The unique contributions of emissions from individual countries, species and sectors to PM_2.5_‐related premature deaths in Europe are characterized at a 0.1° × 0.1° spatial resolution on a monthly basis in 2015. The emission‐induced changes in the sources of PM_2.5_‐related premature deaths are also investigated by comparing source attribution results for 2005 and 2015, during the first 11‐year period of PM_2.5_ regulation in the EU, with the aim of better characterizing (e.g., with finer temporal and spatial details) the sources of regional pollution‐associated health risks and to provide associated implications for environmental policies.

## 2 Data and Methods

### 2.1 Surface PM_2.5_ Concentrations

In situ observations from 972 monitoring sites are used to evaluate the performance of the model simulation of surface PM_2.5_ concentrations in Europe. The observed hourly or daily PM_2.5_ concentrations in 2015 are obtained from the E1a data set, and collected via the EEA Air Quality e‐reporting database (https://discomap.eea.europa.eu/map/fme/AirQualityExport.htm, accessed on: 14 February 2023). The E1a data are validated assessment data annually reported to EEA by each EU member state and have been successfully tested by automated quality control. Information on station type, station area, measurement type, method, equipment as well as data quality are available for each monitoring site via the EEA's air quality portal (https://discomap.eea.europa.eu/App/AirQualityMeasurements/index.html, accessed on: 14 February 2023). Annual mean concentrations are calculated for each monitoring site (with a data capture rate higher than 90%) to compare with the model results. Remote‐sensing derived surface PM_2.5_ concentrations are incorporated into the adjoint simulations to further improve the agreement between the simulation and observation (Text S1 in Supporting Information S1). We use the latest high‐resolution (0.01° × 0.01°) satellite‐based estimates (V5.GL.02) from van Donkelaar et al. (2021) for the base simulation year of 2015. Combining satellite retrievals of aerosol optical depth, chemical transport modeling, and ground‐based measurements, these hybrid PM_2.5_ estimates exhibit general consistency with ground‐based observations. After incorporating the satellite data, the simulated site‐averaged annual mean PM_2.5_ concentration in Europe increases from 12.77 to 14.08 μg m^−3^, which is closer to the observed level (14.98 μg m^−3^), and the *R* ^2^ between the simulated and observed PM_2.5_ concentrations increases from 0.34 to 0.80. Detailed evaluations of simulated PM_2.5_ exposure in Europe are provided in Text S2 in Supporting Information S1.

### 2.2 GEOS‐Chem Forward Model

A nested‐grid capability of the GEOS‐Chem CTM (http://www.geos-chem.org, accessed on: 11 October 2022) is used to simulate the ambient concentrations of aerosols over Europe. The model is driven by assimilated meteorology from the Goddard Earth Observing System (GEOS‐FP) of the NASA Global Modeling and Assimilation Office, which are down‐sampled to a resolution of 0.25° × 0.3125° for the European domain (32.75°–61.25°N, −15°–40°E). Fourty‐Seven vertical layers are included in the model, extending from the surface to 0.01 hPa. To better estimate aerosol concentrations, a new Secondary Organic Aerosol (SOA) scheme is incorporated into the model following Nault et al. (2021) and Nawaz et al. (2021). Other gas‐phase chemistry and aerosol treatments are described in Text S3 in Supporting Information S1. PM_2.5_ is calculated as the total mass of aerosol‐phase sulfate (${SO}_{4}^{2-}$), nitrate (${NO}_{3}^{-}$), ammonium (${NH}_{4}^{+}$), organic carbon (OC), black carbon (BC), SOA, and fine mode mineral dust (aerodynamic diameter less than 1.8 μm). Chemical boundary conditions are provided by a global simulation at a horizontal resolution of 2° × 2.5°, and updated in the nested‐grid region every 3 hours. The base year of the simulation is 2015, when the air quality standards for PM_2.5_ had been introduced for over 10 years.

### 2.3 Emissions

A newly released anthropogenic emission inventory in support of Hemispheric Transport of Air Pollution (HTAPv3 mosaic, https://edgar.jrc.ec.europa.eu/dataset_htap_v3, accessed on: 11 October 2022) is used in the model, obtained from Emissions Database for Global Atmospheric Research. The emission inventory includes monthly emissions of SO_2_, NO_*x*_, carbon monoxide (CO), NMVOCs, NH_3_, PM_10_, PM_2.5_, OC, and BC at the global scale, with a resolution of 0.1° × 0.1° covering the period 2000–2018. In Europe, the HTAPv3 mosaic emissions are from European Monitoring and Evaluation Program—Copernicus Atmosphere Monitoring Service regional inventory (CAMS‐REG, v5.1), built from officially reported emission data provided to Centre of Emission Inventory and Projection (CEIP) by each member state. The inventory covers eight main sectors including shipping, aviation, energy, industry, ground transport, waste, agricultural, and residential emissions. Each main sector is further divided into several detailed sectors (Table S1 in Supporting Information S1), which provides comprehensive information on the sources of air pollutants. NMVOC emissions are lumped into model‐ready emissions for the GEOS‐Chem (Text S4 in Supporting Information S1) and anthropogenic emissions of SOA precursors (SOAP) are calculated following Nault et al. (2021) as described in Text S5 in Supporting Information S1. In addition to anthropogenic emissions, emissions from biogenic (Guenther et al., 2006), biomass (van der Werf et al., 2010), dust (Zender et al., 2003), lightning NO_*x*_ (L. T. Murray et al., 2012), soil NO_*x*_ (Hudman et al., 2012), as well as other natural sources are also including in the model calculation.

### 2.4 Adjoint Sensitivity Calculation

**Figure 1:**
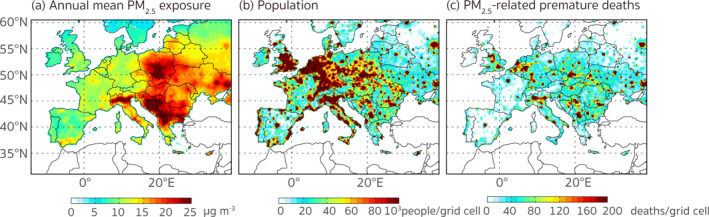
The spatial distributions of the (a) annual mean PM_2.5_ exposure, (b) population, and (c) PM_2.5_‐related premature deaths in 2015.

The GEOS‐Chem adjoint (Henze et al., 2007) v35n is used for sensitivity analyses in the European domain, with the same model resolution and processes as in the forward model described in Section 2.2. Sensitivity analyses begin with the definition of a response (the cost function, $J_{{PM}_{2.5}}$); in this study this is defined as the total number of PM_2.5_‐related premature deaths from COPD, IHD, LRO, LC, T2D, and stroke in all the European countries listed in the Global Health Data Exchange (GHDx, https://ghdx.healthdata.org/, accessed on: 11 October 2022) over the targeted receptor region (shaded areas in Figure 1) in 2015 (Text S6 in Supporting Information S1). After one year of spinning up of the forward model, twelve 1‐month adjoint simulations are conducted in 2015, in which gradients of the cost function with respect to emissions of each PM_2.5_ precursors in each grid cell and month are calculated. With these so‐called adjoint sensitivities $\lambda_{E}$, km‐scale (0.1° × 0.1°) contributions from emissions of 6 species and 16 sectors defined in the HTAP v3 emission inventory can be quantified at a monthly basis (Text S7 in Supporting Information S1). By comparing the different emission contributions, we further quantify the emission induced changes in PM_2.5_‐related health impacts in Europe during 2005–2015, which is the first stage of EU PM_2.5_ regulation. To obtain similar results as these would require 1,403,136 sensitivity simulations if using forward‐modeling or other source‐oriented methods. Though the time required by a single adjoint sensitivity calculation might be approximately 10 times the computational cost of a single forward run (Henze et al., 2007), the adjoint approach can still be more than 11,692 times faster than the forward‐modeling based calculations.

## 3 Results

### 3.1 Premature Deaths Attributable to PM_2.5_ Exposure

We present the calculated spatial distributions of the annual mean PM_2.5_ exposure, population, and PM_2.5_‐related premature deaths in Figure 1. The total number of PM_2.5_‐related premature deaths (i.e., the cost function) are calculated to be 449,813 out of a population of 598.97 million over the receptor region in 2015. Considering the uncertainty introduced by the forward and adjoint model calculations as well as the data and method chosen for the health assessment, we calculated lower and upper bounds for these health impacts which we discuss in more detail in Section 3.5. Table S2 in Supporting Information S1 lists estimates of total PM_2.5_‐related premature deaths in Europe obtained from other recent model studies. Our estimate, although slightly lower than that of Lelieveld et al. (2019) in 2015, agrees well with the magnitudes of model calculations in these studies. The spatial distribution of the estimated premature deaths is also consistent with previous literature studies (e.g., Im et al., 2018). As Figure 1 shows, most deaths were from populated regions (e.g., central Europe) or areas where the PM_2.5_ levels or the baseline mortality rates are high (e.g., eastern Europe, Figure S3 in Supporting Information S1). Compared to western and central Europe, inhabitants in eastern European countries experienced higher risks associated with PM_2.5_ pollution, and the mortality per grid cell exhibited much higher values. The main causes of the estimated health risks were IHD and stroke, accounting for 44.8% and 23.5%, respectively, to the total PM_2.5_‐related premature deaths over the receptor region.

### 3.2 Source Attribution of PM_2.5_‐Related Premature Deaths

Using the adjoint sensitivities ($\lambda_{E}$, Section 2.4), contributions of anthropogenic emissions from distinct species and sector groups to total PM_2.5_‐related premature deaths over the receptor region are calculated at the 0.1° × 0.1° resolution of the HTAPv3 emission inventory. Table S3 in Supporting Information S1 summarizes the annual source contributions aggregated over various precursor species and sector groups. Anthropogenic emissions of NO_*x*_, NH_3_, SO_2_, OC, BC, and SOAP over the nested model domain are calculated to contribute 265,328 PM_2.5_‐related premature deaths in the receptor region in 2015, accounting for approximately 59.0% of the total premature deaths from all PM_2.5_ in the region. The results suggest that a majority of the PM_2.5_‐related health risks in Europe were associated with domestic emissions of these species within Europe, yet there is still a large proportion of contributions from other anthropogenic, natural or external sources. In this section, we mainly focus on the source attribution of the domestic anthropogenic contributions. The contributions from other emission sources are discussed in Section 3.3 below.

#### 3.2.1 Source Contributions to PM_2.5_‐Related Premature Deaths

**Figure 2:**
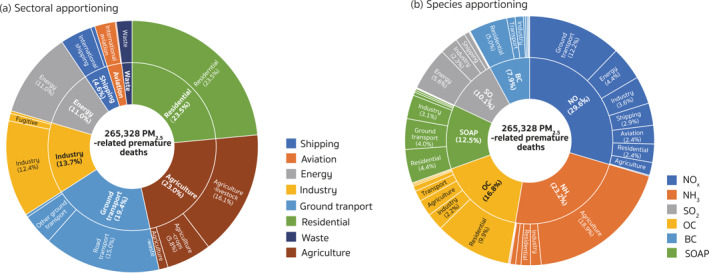
Annual source apportionment of the PM_2.5_‐related health risks contributed by the anthropogenic emissions within Europe. The pie charts indicate the (a) sectoral and (b) species apportioning, respectively, of the PM_2.5_‐related premature deaths over the nested European domain in 2015. The number in parentheses is the percentage of the contributions from each category in the total PM_2.5_‐related premature deaths induced by anthropogenic emissions within Europe.

Figure 2a presents the relative contribution from each sector group to the total PM_2.5_‐related premature deaths attributable to the anthropogenic emissions of NO_*x*_, NH_3_, SO_2_, OC, BC, and SOAP within the nested model domain. As the source attribution results suggest, residential, agricultural, and ground transport emissions were the major sources of the regional PM_2.5_‐related health risks, accounting for 13.9%, 13.6%, and 11.4% of the total burden of PM_2.5_‐related premature deaths in Europe in 2015. The results are consistent with earlier works in Europe (e.g., Crippa et al., 2019; Lelieveld et al., 2015; Silva, Adelman, et al., 2016), that found that annual PM_2.5_ concentrations and corresponding health effects stemmed mainly from the agricultural and residential sectors, followed by the transport sector. In our estimate, however, residential contributions are higher than the contributions from agricultural emissions, making residential emissions the largest anthropogenic source category. This difference between our study and previous works can be explained by the different treatments of SOA. Limited by the availability of emission inventories of SOA precursor gases, no explicit treatment of anthropogenic SOA was considered in previous calculations, whereas our study incorporates the newly developed SOA scheme (Nault et al., 2021) into the model and includes SOA contributions when estimating PM_2.5_ concentrations. The inclusion of SOA leads to increased contributions from sectors with large VOC emissions, like residential, transport, and industry. Agriculture‐livestock and road transport emissions are calculated to be the major sources of agricultural and ground transport contributions, respectively, contributing 69.9% and 77.4%, respectively to the sector contributions.

**Figure 3:**
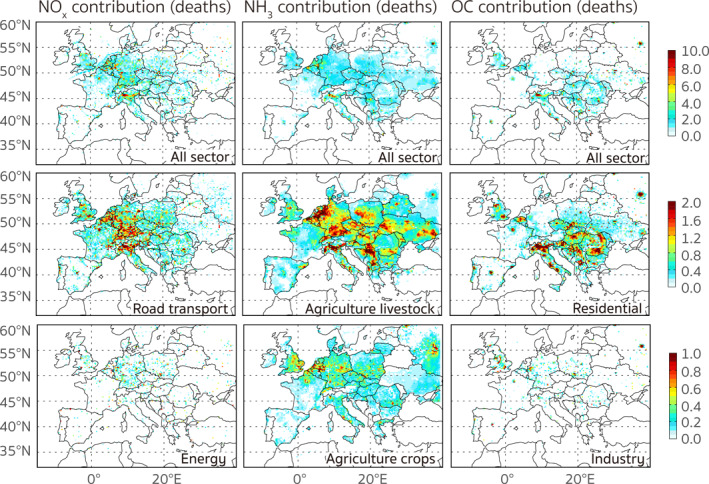
The spatial distributions of the annual contributions from anthropogenic emissions of NO_*x*_, NH_3_, and organic carbon to the total PM_2.5_‐related premature deaths over the receptor region. The first row shows the total contributions from each species and the subsequent rows show the spatial distributions of the two sectors with the largest contributions for each species. The spatial distributions are presented at the fine resolution (0.1° × 0.1°) of the HTAPv3 emission inventory.

For species contributions (Figure 2b), emissions of NO_*x*_, NH_3_, and OC were the top 3 ranked contributors, making up 29.6%, 23.2%, and 16.8%, respectively of the total anthropogenic contributions within Europe. Most NO_*x*_‐contributed PM_2.5_‐related premature deaths were associated with the transport emissions, with ground transport, shipping, and aviation making up 59.0% of the total NO_*x*_ contributions. Agricultural activities (e.g., crops, livestock, and waste) were related with 81.7% of the NH_3_ contributions and 59.0% of the OC contributions were from residential sources. Though energy and industry emissions might not play as dominant a role as the other sectors, they were still the second largest sources of NO_*x*_ and OC contributions, respectively, and made up 79.2% of the contributions from SO_2_ emissions. To further analyze the source regions of the contributions associated with the anthropogenic emissions within Europe, Figure 3 displays the spatial distributions of the major species contributions at the resolution of the HTAP v3 emissions (0.1° × 0.1°). With the adjoint calculated fine‐resolution sensitivity, source regions of the contributions can be easily identified even from individual point sources and transport systems. As is shown in Figure 3, road transport contributions originated mainly from central European countries (e.g., Benelux, Germany, and Italy), while residential contributions were concentrated in southern and southeastern European countries (e.g., Italy, Hungary, and Romania). Contributions from agricultural sources were less spatially confined, exhibiting high values in northwest and southeast Germany, north Italy, Czechia, Hungry, Serbia, Poland, and Ukraine. To better understand the roles of different countries in influencing the PM_2.5_‐associated health risks in Europe, we discuss more details about the contributions at the country level in Section 3.3. Compared to the contributions from transport, agricultural, and residential sources, contributions of emissions from industry and energy sectors were more from point sources, originating mainly from the United Kingdom (UK), Germany, Poland, Romania, and the European part of Russia.

#### 3.2.2 Monthly Source Attribution

**Figure 4:**
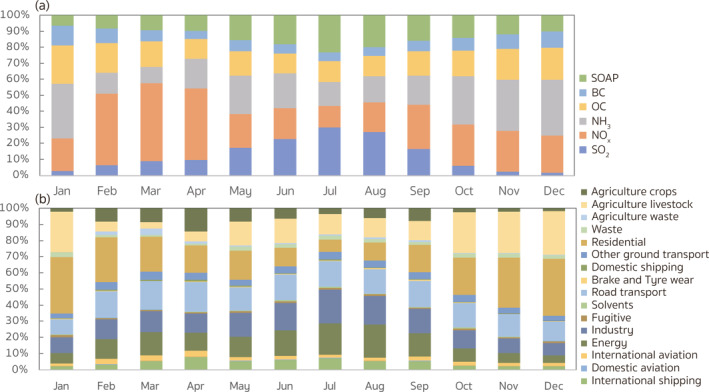
Monthly variations of (a) species and (b) sectoral contributions to the PM_2.5_‐related health risks aggregated over the nested model domain. Normalization for each month is done by dividing each source category contribution by the total anthropogenic contribution during the same month. The colors in each bar match the ordering provided in the legend.

In addition to the source apportionment at the annual time scale, we also characterize the monthly changes in contributions from different species and sectoral emission sources in Figure 4. Given the seasonality of the meteorology, emissions, as well as the physical and chemical processes in the atmosphere, the monthly source attribution results provide additional details that enable policy‐makers to make better decisions compared to those made solely based on annual results. As Figure 4a shows, the monthly contributions exhibited different changes among various species. Emissions of OC and BC were associated with more damages to the public health in winter (December, January, and February), contributing 215.5% and 259.2%, more premature deaths, respectively, than those in summer (June, July, and August). The results are consistent with the seasonal changes in residential contributions (Figure 4b), which accounted for over 70% of the OC and BC contributions in winter, and only 20%–40% of those in summer. As Text S7 in Supporting Information S1 explains, the contributions are determined by two parameters: the adjoint sensitivities and emissions. To illustrate the seasonal changes of source contributions, Figure S2 in Supporting Information S1 displays the normalized monthly variations of mean sensitivities, and total emissions for each species and sector over the receptor region. In addition to the increased residential emissions, the sensitivities of the PM_2.5_‐related health risk to the OC and BC emissions in winter also exhibited values 37.4%–39.2% higher than those in summer, further contributing to the seasonal differences. In contrast, emissions of SO_2_ and SOAP had more adverse impacts on the public health in summer, contributing to 3.9 and 1.2 times, respectively, more premature deaths than those during wintertime. Similar patterns are found in the contributions of emissions from the energy and industry sectors, where approximately 78.1% of the SO_2_ contributions originated. In Figure S2 in Supporting Information S1, the emissions from energy and industry sector show similar decreases, though relatively smaller, as the residential emissions in summer. Along with their major sources, the emissions of SO_2_ and SOAP in summer also exhibited values 23.6% and 19.4% lower than those in winter, respectively. In contrast, the summertime sensitivities for SO_2_ and SOAP emissions were 326.4% and 73.7% higher, respectively, than those during wintertime, as the photo‐chemical oxidation needed for the formation of sulfate and SOA increases. The results suggest that the influence of the sensitivity changes overcame that impacts of the emission changes, which determined the seasonality of the contributions from SO_2_ and SOAP emissions.

Figure 4a also shows that contributions from NO_*x*_ emissions exhibited peak values during February to April, accounting for 47.2% of the annual total contributions from NO_*x*_ emissions. As discussed in previous studies, the wintertime sensitivities for NO_*x*_ and NH_3_ are usually higher due to the favorable formation conditions of ammonium nitrate (Guo et al., 2019; Nawaz et al., 2021). In this study, even BC, which is barely involved in chemical reactions, exhibited higher sensitivities during the early spring (Figure S2 in Supporting Information S1), indicating that the meteorological conditions (e.g., low surface wind speeds) can be more beneficial for the accumulation of surface aerosols during that time. In addition to the increased NO_*x*_ sensitivities, NH_3_ emissions show significant increases during the same period as the emissions from agricultural crops and waste sources, which contributed to 66.5%–80.8% of all NH_3_ emissions during February to April, increased before the start of the growing season. The increased NH_3_ emissions and NO_*x*_ sensitivities provide extremely favorable conditions for the formation of ammonium nitrate, resulting in relatively large contributions of emissions from NO_*x*_‐rich sector groups.

#### 3.2.3 Country‐Level Source Attribution

**Figure 5:**
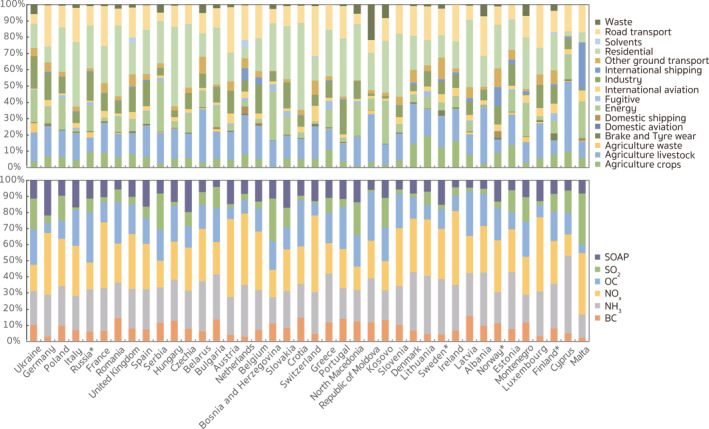
Relative contributions from emissions associated with specific sectors and species to the total health burden from anthropogenic emissions from each European country (region). Countries (regions) are listed in descending order from left to right according to their contributions to the total PM_2.5_‐associated premature deaths over the receptor region in 2015. The name followed by an asterisk indicates a country that only lies partially within our nested model domain. The colors in each bar match the ordering provided in the legend.

In this section, we examine the contributions from anthropogenic emissions at the country level in order to characterize the roles of different countries in influencing the PM_2.5_‐associated health risks in Europe. Tables S4 and S5 in Supporting Information S1 list the sectoral and species contributions, respectively, from each European country in 2015, while corresponding relative contributions to the total nationwide contributions are displayed in Figure 5. Ukraine, Germany, Poland, Italy, Russia (though only partially included in the model calculation, with approximately 30% of its total population), and France were the top six source countries, contributing to over 56.7% of the total anthropogenic PM_2.5_‐related premature deaths within Europe; this emphasizes the importance of regulating anthropogenic sources in these key source countries. However, as Figure 5 shows, the sectoral and species contributions exhibited strong variability at the country level. For western and central European countries (e.g., Germany, France, Benelux, Switzerland), ground transport emissions were the dominant anthropogenic sources of PM_2.5_‐related health risk, accounting for 25%–40% of the nationwide contributions. Consequently, anthropogenic NO_*x*_ emissions were the most important sources of the premature deaths, making up over 35% of the nationwide contributions. In Mediterranean and Eastern countries (like Italy, Spain, Poland, and Romania), the anthropogenic contributions were mainly from the residential sector emissions, which can even make up over 50% (e.g., Croatia 53.2%) of the total deaths contributed by the nationwide emissions. Correspondingly, in these countries the influence of carbonaceous aerosol emissions is substantial. Due to targeted pollution regulations, emissions from energy and industry sectors were usually not the dominant contributors in European countries, but for countries which were greatly influenced by point sources (e.g., Serbia), they still accounted for large proportions of the nationwide contributions. For example, energy contributions made up about one third of the total nationwide PM_2.5_‐related premature deaths in Serbia, which was 1.2 and 5.7 times higher than the contributions from its domestic residential and ground transport emissions.

**Figure 6:**
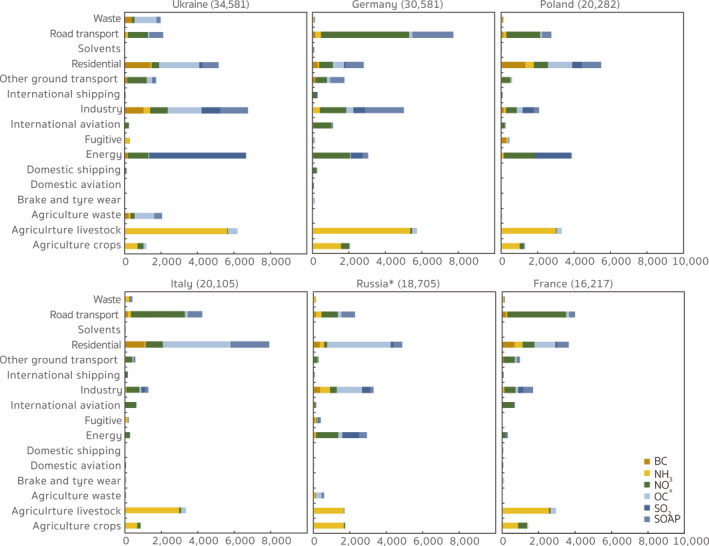
Sector‐specific anthropogenic contributions from Ukraine, Germany, Poland, Italy, Western Russia (European parts), and France. The number in parentheses in the title refers to the total PM_2.5_‐related premature deaths contributed by the anthropogenic emissions from each country (region). The name followed by an asterisk indicates a country that only lies partially within our nested model domain.

In Figure 6 we present more detailed annual source appointments of the nationwide contributions from the top six ranked contributing countries in 2015. The results allow us to identify which species from which emission sector in these countries contributed most to the adverse impacts on public health in Europe, providing more practical implications for policy making. For example, emissions from Ukraine contributed 34,581 premature deaths in Europe, most of which were contributed by emissions from industry (19.6%), energy (19.3%), agriculture livestock (18.0%), and residential (14.9%) sources. Emissions of OC, SO_2_, and NH_3_ were the dominant sources of industry, energy, and agriculture livestock contributions, respectively, accounting for 27.5%, 79.4%, and 90.6%, respectively of each sector contributions. In contrast, emissions from ground transport (25.3%), agriculture livestock (18.8%), and industry (16.5%) sectors were the main sources of the premature deaths contributed by anthropogenic emissions in Germany. Unlike in Ukraine, SOAP emissions made up most (42.8%) of the industrial contributions from Germany, suggesting that the local industrial structure might lead to large differences in the country‐level source contributions, even from the same sector. Differences across source contributions can also be found within the energy sector. In Poland, emissions from residential and energy sectors were associated with 27.0% and 19.1%, respectively of the total nationwide contributions. 50.1% of the energy contributions were from SO_2_ emissions and 44% of those were from NO_x_ emissions, which was quite different from Germany and Ukraine where the dominant contributions in the energy sector came from NO_*x*_ (66.0%) and SO_2_ (79.4%) emissions, respectively. For premature deaths contributed by emissions from Italy, 60.5% were from the residential and road transport sectors. Similar source attribution results are found in France, where emissions from the road transport and residential sectors made up 24.7% and 22.6%, respectively of the nationwide contributions. For all six countries, the source attributions of contributions from these two sectors were more consistent compared to those from industry and energy sources, with NO_*x*_ emissions making up a majority of the nationwide transport contribution and OC emissions making up a large proportion of the nationwide residential contributions.

### 3.3 Local and Regional Contributions to the PM_2.5_‐Related Premature Deaths

In Section 3.2, we quantify the contributions of anthropogenic emissions from each individual country, species, and detailed sector to PM_2.5_‐related premature deaths within Europe. In this prior analysis, we only consider the anthropogenic sources within our nested European domain and thus the results explain only 59% of the total estimated premature deaths. The remaining 41% of the premature deaths are attributable to emissions from natural sources within Europe and emissions from outside Europe. Those sources aside, contributions from domestic anthropogenic emissions can be largely influenced by strong transboundary transport among European countries. The study of Crippa et al. (2019) suggested that transboundary air pollution contributed 25%–75% to PM_2.5_ pollution in European counties. Here we discuss the contributions to PM_2.5_‐related premature deaths from extra‐regional sources (Section 3.3.1) as well as the redistribution of the PM_2.5_‐related premature deaths contributed by the anthropogenic emission within Europe (Section 3.3.2). The results can help us learn about the premature deaths attributable to sources other than the domestic European anthropogenic emissions discussed in the previous section. This provides further understanding of the limitations of local policies in reducing the pollution related regional health risks within Europe.

#### 3.3.1 Contributions From Extra‐European Sources

To understand the influence of sources of PM_2.5_‐related premature deaths beyond anthropogenic emissions within Europe, we consider contributions from extra‐regional sources in this section. For the nested simulation, the boundary conditions are generated by a first, global simulation at a horizontal resolution of 2° × 2.5°, the inputs from which can be considered as the influence of sources from areas outside the nested‐grid region, and are updated every 3 hours in the simulation. Therefore, we conduct a perturbation experiment by reducing all inputs of the boundary conditions by 20% (BC‐20), and the contributions of the extra‐regional emissions are calculated based on the differences between the estimations in the base model run (BASE) and the BC‐20 scenario run. To quantify the effects of a 20% decrease in boundary conditions, we only apply the satellite downscaling to the premature death estimation in both BASE and BC‐20 computations, since the satellite rescaling would correct the bias induced by the perturbation. The contribution resulting from extra‐European emissions in each grid cell is then estimated to first order by multiplying the corresponding difference induced by the 20% perturbation by five.

**Figure 7:**
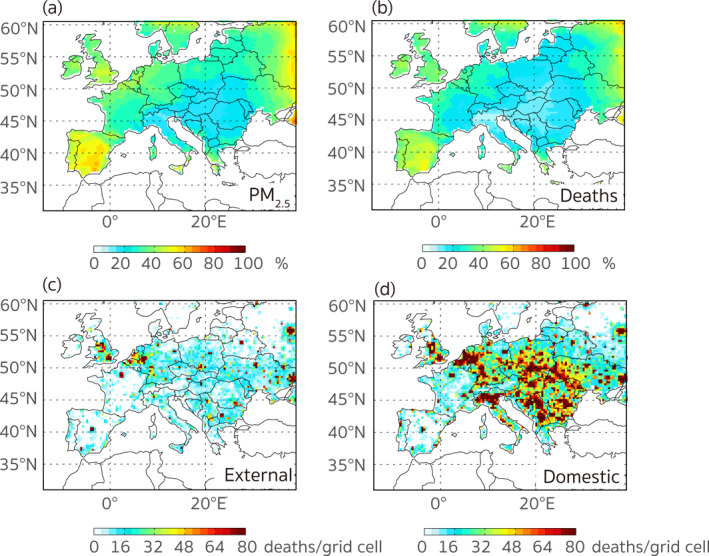
The relative contributions of the extra‐regional emissions to (a) the surface PM_2.5_ concentrations (population‐weighted) and (b) the PM_2.5_‐related premature deaths in Europe. (c, d) Are the calculated PM_2.5_‐related premature deaths contributed by emissions outside and within the nested‐grid region.

In Figures 7a and 7b we present the relative contributions from external sources to population‐weighted PM_2.5_ concentrations and PM_2.5_‐related premature deaths in 2015. The perturbation results suggest that the impact of extra‐regional emissions decreased from the near‐boundary areas to the central regions, and the extra‐regional emissions contributed to 31.4% of the PM_2.5_ exposure and 25.1% of the premature deaths, respectively, averaged over the European region. The calculated contributions of extra‐regional sources to PM_2.5_‐related health burden in Europe are comparable to those (∼22%) found in previous studies (e.g., Anenberg et al., 2014; Crippa et al., 2019; Im et al., 2018; Liang et al., 2018). This suggests that PM_2.5_ and its associated health burden in Europe were mainly attributable to domestic sources, yet the extent of domestic influences varied due to several factors including the definition of the receptor region, the inclusion of natural components, the target year, the perturbation method, and differences in model‐setups (e.g., resolution, physical and chemical mechanisms) used in the calculation. Contributions from extra‐regional emissions (Figure 7c) are calculated by multiplying the estimated premature deaths (Figure 1c) by the relative contributions as displayed in Figure 7b. In our receptor region, the extra‐regional emissions contributed approximately 113,087 premature deaths, leading to more adverse impacts in hotspots near the boundaries (e.g., south UK, Benelux, and east Ukraine). The premature deaths caused by sources within the nested‐grid region (Figure 7d) are then obtained according to the differences between the estimated total premature deaths and the calculated external emission contributed deaths. Thus, domestic anthropogenic emissions of NO_*x*_, NH_3_, SO_2_, OC, BC, and SOAP, as mentioned in Section 3.2, lead to 265,328 premature deaths, accounting for approximately 78.8% of the total contributions of emissions within nested domain. The previous literature emphasizes that natural sources (e.g., dust) also contributed strongly to mortality, making up about one‐sixth of global air pollution induced premature deaths (Lelieveld et al., 2015). In this study, we consider fine mode dust particles as a component of PM_2.5_, and assume that they are equally toxic as other fine particulate matter species (e.g., ${SO}_{4}^{2-}$, ${NO}_{3}^{-}$, ${NH}_{4}^{+}$, OC, BC). The nested‐grid region includes not only large ocean areas but also northern Africa and even parts of the Middle East where natural sources contribute 15%–92% of the local premature mortality. Therefore, the remaining deaths other than those contributed by domestic anthropogenic sources can be largely attributable to the contributions of natural sources including dust, sea salt or large‐scale biomass burning, as well as to the initial pollution conditions inherited from the previous months, accounting for 15.9% of the estimated premature deaths in Europe, or 21.2% of the total contributions of emissions from within the domain.

#### 3.3.2 The Redistribution of the Local Contributions Within Europe

**Figure 8:**
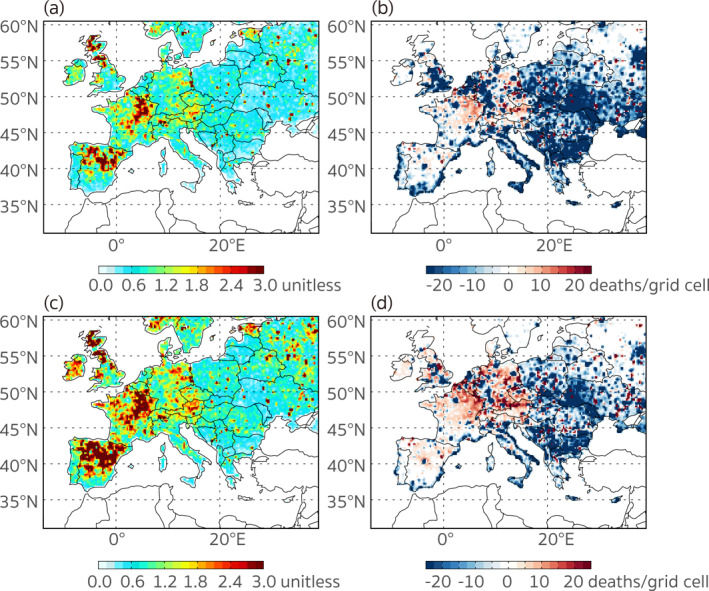
The spatial distributions of (a) the contribution ratio (the contribution of anthropogenic emissions to premature deaths anywhere in Europe divided by the premature deaths occurring in each grid cell.) and (b) the absolute difference between the anthropogenic emission contributions to premature deaths anywhere in Europe and the estimated premature deaths in each grid cell. (c, d) Are similar to (a, b), but the estimated actual premature deaths are replaced by those caused by emissions only within Europe.

There are complex links between emissions and PM_2.5_ concentrations, since the pathways of emitted air pollutants depend on not only sources, but also meteorological conditions, geographical features, and their chemical properties. In addition to the pollution level, PM_2.5_‐associated premature deaths in a specific region are also related to the exposed population and even the medical conditions that determine the disease mortalities, which raises the complexity of the relationship between the magnitude of emission and the number of premature deaths that occur locally. To examine this further, we define the contribution ratio (CR) as the contribution of anthropogenic emissions to premature deaths anywhere in Europe divided by the premature deaths occurring in each grid cell. Figure 8 displays the spatial distributions of CRs and the absolute differences between the two types of premature deaths in 2015. Here we consider the actual premature deaths as the total estimated deaths in Figures 8a and 8b and the estimated deaths excluding the influence of the extra‐regional emissions (Figure 7d) in Figures 8c and 8d, respectively. The former provides us an overall view of the health risks contributed by local emissions relative to the total health burden experienced by the local population, and the latter helps us better understand the redistribution of anthropogenic contributions controllable within the nested‐grid region. As is shown in Figure 8a, the emissions from some western and central European regions caused more premature deaths than those that occurred locally. The “over‐contributing” regions, that is, those whose emissions contributed to more premature deaths than were incurred locally (Figure 8b), mainly occurred in northeast Spain, central UK, northeast France, Luxembourg, east Germany, and Austria, where the estimated number of premature deaths from anthropogenic emissions exceeded the number of deaths that occurred locally by a factor of three. In a sense, these regions are net “exporters” of air pollution health impacts, given that their emissions cause more premature deaths than they alone experience. In contrast, most eastern European countries, except for regions where large point sources were located, suffered from more premature deaths than were caused by their own throughout Europe; they are net “importers” of health damages in this sense. For example, the anthropogenic emissions from Greece only caused an estimated 2,224 premature deaths, while 7,356 premature deaths occurred there. After the impacts from extra‐regional sources are excluded in Figures 8c and 8d, the redistribution of the contributions by sources within Europe exhibits a distinct pattern of the adverse pollution‐related health risks being transferred from the west to the east. The most “over‐burdened” counties/regions (with a within‐region CR of less than 0.42, Table S6 in Supporting Information S1) included Greece, Bulgaria, Andorra, and Cyprus. The results are consistent with the calculation of Crippa et al. (2019) who indicated that the PM_2.5_ concentrations in these regions could be caused more by extra‐regional sources than the domestic emissions.

The local PM_2.5_‐related premature deaths are determined by the population, mortality rates of pollution‐associated health outcomes, and the exposure level (Text S6 in Supporting Information S1). As displayed in Figure 1a and discussed in previous works (e.g., Ciarelli et al., 2019; Kiesewetter et al., 2015), eastern European countries can be characterized as pollution hotspots where the population was exposed to higher concentrations of PM_2.5_ than in western or central European countries. The relative health risk was thus higher according to the exposure response relationship following the GBD 2019 study (C. J. L. Murray et al., 2020), which means a larger proportion of the premature deaths would be attributed to PM_2.5_ exposure. Additionally, the baseline mortality rates associated with pollution‐related diseases (IHD, COPD, LRI, LC, T2D, and STROKE, Figure S3 in Supporting Information S1) were higher in eastern European countries. Taking Ukraine as an example, the average death rate across the six diseases was 1,025 deaths per 100K population in 2015 according to the GBD results, which was 3.5 times the number in France (∼295 deaths per 100K population). The high mortalities might be related to poor medical conditions or low socio‐economic status. A concern is that the high mortalities result in more premature deaths responding to per unit increases in PM_2.5_ concentrations, further aggravating the detrimental health impacts of pollution. As indicated in previous source appointments (e.g., Crippa et al., 2019; Im et al., 2018) and our results (see Sections 3.2.1 and 3.2.3), eastern European countries (like Ukraine, Poland, and Romania) were estimated to be among the major sources of the burden of pollution‐related disease in Europe due to the burning of solid fuels for domestic heating and industry. However, according to the discussions above, we find that people living in some of these regions experienced even greater harmful air pollution effects than their local sources caused, since they were not only more susceptible to the adverse health effects of severe PM_2.5_ pollution but also received PM_2.5_ emanating from western and central Europe. This redistribution further increases the heterogeneity of the pollution related health risks in Europe and might lead to larger social inequalities in health and other socio‐economic aspects.

### 3.4 Emission‐Induced Contribution Changes Between 2005 and 2015

As discussed above, domestic anthropogenic emissions are the most important sources of PM_2.5_‐related health risks in Europe. To tackle air pollution and protect human health and the environment, the EU has implemented a comprehensive clean air policy during the past two decades. Air quality standards (e.g., Directive 2008/50/EC) and national reduction commitments (e.g., Directive (EU) 2016/2286) for air pollutants (e.g., SO_2_, NO_*x*_, NMVOCs, NH_3_, and PM_2.5_) were introduced and updated in different stages. In this section, we consider the first stage of the PM_2.5_ regulation in Europe, which lasted from 2005, when the cap of 25 μg^−3^ for annual mean PM_2.5_ concentrations was first put forward, until 2015, when the limit value was met (European Commission, https://environment.ec.europa.eu/topics/air/air-quality/eu-air-quality-standards_en, accessed on: 11 August 2022). We quantify how such policy‐induced decreases in aerosol and its precursor emissions contributed to health benefits within Europe. To exclude the influence of other factors (e.g., meteorology, natural sources, and socio‐economic conditions), we assume that the adjoint sensitivity for each species in 2005 is consistent with that in 2015, and calculate the corresponding contributions of emissions from each individual country, species, and sector according to Text S7 in Supporting Information S1.

**Figure 9:**
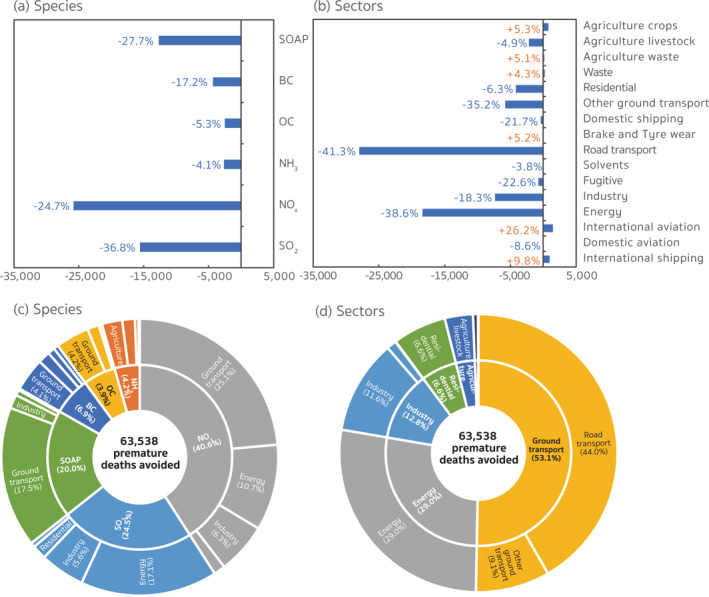
Changes in the PM_2.5_‐related premature deaths contributed by (a) species and (b) sectoral emission changes during 2005–2015 over the receptor region. The percentages indicate the relative changes in 2015 compared to the number in 2005. Pie charts in panels (c and d) show the proportions of the species and sectoral contributions in the total premature death changes contributed by the anthropogenic emission reductions. The number in parentheses is the percentage of the contribution changes from each category in the total anthropogenic emission‐induced changes in PM_2.5_‐related premature deaths within Europe.

Figure 9 shows the total contribution changes for each species and sectoral source within Europe between 2005 and 2015. Reductions in anthropogenic emissions during this 11‐year period can account for 63,538 fewer PM_2.5_‐related premature deaths. Avoided deaths were primarily attributable to decreased contributions from NO_*x*_, SO_2_, and SOAP emissions, accounting for 40.6%, 24.6%, and 20.0%, respectively, of the total decreases in the premature deaths. Most sectoral sources contributed to the decreases, albeit to varying degrees. Consistent with the large reductions in NO_*x*_ contributions, ground transport emission reductions contributed more than half (53.1%) of the total deaths avoided by the emission control. Decreases in energy and industrial emissions resulted in 29.0% and 12.8%, respectively, of the total emission‐induced decreases in premature deaths, which were the other two major sources of the health benefits. SO_2_ contributions exhibited the second largest decreases (36.8%) during 2005–2015, 69.6% of which were from decreased emissions from energy sources. In contrast to the large decreases in contributions from ground transport and energy emissions, premature deaths from residential and agricultural sources, which were the largest two sources of PM_2.5_‐related premature deaths in Europe (Figure 2) in 2015, exhibited very slight changes, with corresponding decreases accounting for only 6.6% and 2.0%, respectively of the total emission‐induced decreases in the premature deaths. Further, contributions of emissions from agriculture waste burning and agriculture crops even increased by 5.1% and 5.3%, respectively in 2015 compared those in 2005. Similar increases occurred in contributions from sources like waste, international shipping and aviation, among which premature deaths contributed by international aviation emissions increased most (26.2%) in 2015, leading to 1,453 more deaths compared to the beginning of the first stage. It should be noted that we consider only the impacts of anthropogenic emission changes on the health benefits here. The GBD results (https://vizhub.healthdata.org/gbd-results/, accessed on: 11 October 2022) suggested that the total number of deaths from COPD, IHD, LRO, LC, T2D, and stroke decreased by 383,601 (330,238–445,449) in Europe during 2005–2015, and the changes accounted for 9.5% of the total deaths in 2015. If the demographic changes are considered, the values of relative emission‐induced changes, depicted as the total PM_2.5_‐related premature deaths in 2015 minus those in 2005, should be smaller than what we present here. However, those differences are likely very small compared to those induced by health impact assessment uncertainty, which we discuss with more details in Section 3.5.

**Figure 10:**
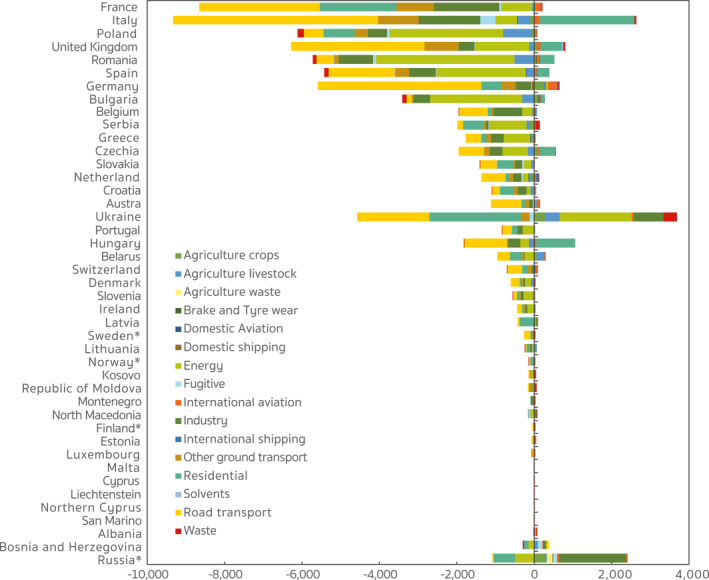
The attribution of country (region)‐specific changes in the PM_2.5_‐related premature deaths contributed by anthropogenic emissions within the receptor region during 2005–2015. The name followed by an asterisk indicates a country that only lies partially within our nested model domain.

As for changes at the country level, the results displayed in Figure 10 suggest that nearly all European countries decreased their contributions to the PM_2.5_‐associated health risks in the first stage of emission controls, though the magnitude and attribution of changes varies widely. For example, anthropogenic emission reductions in France, Italy, and Poland were the leading contributors to health benefits, accounting for 13.3%, 10.5%, and 9.5%, respectively, of the total deaths avoided in Europe. Emission reductions in road transport, residential, and industry sectors drove the bulk of the calculated decreases in France. In Poland, however, those decreases were dominated by energy emission reductions, accounting for 48.3% of the total decreases nationwide. In Italy, premature deaths contributed by road transport emissions exhibited significant decreases, but this benefit was largely offset by increased contributions from Italian residential emissions which led to 2,462 more deaths in 2015 compared to the number in 2005. Similar increases in contributions from residential emissions occurred in many other countries (e.g., United Kingdom, Romania, Spain, Bulgaria, Czechia, and Hungary), which explains the relatively small change in the total contribution of residential emissions during the study period. Figure 10 also indicates that while in general there were large decreases, there were still some contributions that exhibited only slight decreases or even increases. For example, premature deaths contributed by energy and industrial emissions increased by 1,856 and 770, respectively in Ukraine, and by 15 and 23, respectively in Latvia, in contrast to the average decreasing trend in Europe. Compared to the contributions from other sources, premature deaths attributed to agricultural activities exhibited very slight decreases, and, for some countries, even large increases, especially for those related to crop production.

### 3.5 Uncertainty Analysis and Limitations

The uncertainty in our analysis arises from several sources: the satellite‐derived PM_2.5_ concentrations used for calculation of PM_2.5_ exposure, the estimation of the health impacts associated with this exposure, the adjoint model calculation of exposure sensitivities to emissions, the application of these sensitivities using a first‐order linear approximation of source contributions, and the magnitude of the emissions themselves. Previous source attribution studies have shown that the estimation of exposure‐associated health impacts is usually the largest source of uncertainty in health impact assessments (Nawaz & Henze, 2020; Nawaz et al., 2021). While we discuss the uncertainties from sources other than this in the following paragraph, we treat them separately and only consider uncertainties caused by the estimation of exposure‐associated health impacts in determining the uncertainty bounds, since the covariance between the health impact calculation and other types of uncertainties remains to be investigated.

We apply satellite downscaling and rescaling (Text S1 and S2 in Supporting Information S1) to the simulated surface concentrations before the values are passed to the adjoint calculation. Artifacts in the calculated exposure can be discerned by comparing the corrected concentrations to the measured PM_2.5_ values. In the selected 972 monitoring sites, the simulated PM_2.5_ concentrations after the satellite correction have a low bias of 6.0% in 2015 (Figure S1b in Supporting Information S1). We assume these biases can then lead to slight underestimation of the exposure and related health impacts. Additionally, the satellite product itself also contributes to the uncertainty, in which the annual mean PM_2.5_ concentrations exhibits overall uncertainties of −8% to +13% in Europe (van Donkelaar et al., 2021), influencing the relative uncertainty in our estimation. Uncertainty in the adjoint model's PM_2.5_ source‐receptor sensitivities can be associated with uncertainties in meteorology as well as the chemical and physical processes represented by the GEOS‐Chem model. Such kinds of uncertainty can be accessed by comparing the original model results (without satellite rescaling and downscaling) to the observations. It should be noted that the evaluated model performance is also coupled with the uncertainty in emissions, which is difficult to separate from the model uncertainties. As Figure S1a in Supporting Information S1 shows, the overall total bias induced by these two factors combined is approximately −2.21 μg m^−3^, which translates into about −15% underestimation in the PM_2.5_ levels and related calculations in Europe. The adjoint model sensitivities are also merely tangent linear gradients, the application of which is likely to be reasonable over a limited range of perturbations (Henze et al., 2007). For OC, BC, and primary species, their response to the emission perturbations is linear, so that errors from a first‐order linear approximation are close to zero. In contrast, for NH_3_, NO_*x*_, SO_2_, and SOA, the first‐order linear approximation neglects higher order sensitivities, giving rise to relatively larger errors in exposure and related health impacts when large emission perturbation occurs. During 2005 to 2015, the overall anthropogenic emissions of NH_3_, NO_*x*_, SO_2_, and SOA changed by −1.3%, −19.3%, −34.9%, and −26.8%, respectively over the studied region. These magnitudes are still within the perturbation range for which first‐order linear approximation is applicable (Henze et al., 2007; Koo et al., 2013). For PM_2.5_, the first‐order sensitivities to emissions of NO_*x*_, NH_3_, SO_2_ and VOCs were calculated to be one or two orders of magnitude higher than the higher‐order sensitivities (Koo, 2011), indicating that the first‐order linear approximation can be considered accurate even though small uncertainties might result from ignoring the impacts of higher‐order sensitivities.

The health impact assessment uncertainty arises from uncertainty in the estimates of population, mortality rates of health outcomes, and the exposure response relationships used to calculate pollution‐related premature deaths from health outcomes. The latter is usually considered to be the major source of uncertainty in the health impact analysis, and is typically used in determining the uncertainty bounds (Lee et al., 2015). Uncertainties in the gridded population data used here from CIESIN (2018) are not explicitly available. However, by comparing the total population of each European country to the estimates provided by the GBD 2019 results (https://vizhub.healthdata.org/gbd-results/, accessed on: 11 October 2022), we can calculate an uncertainty range as the percent difference comparing the model population to both of the GBD bounds, of −20% to +54% for the country‐level population over the studied region. The GBD results provide explicit uncertainty bounds for mortalities and relative risks. By considering the range of all three of these factors, we estimate that the total number of PM_2.5_‐related premature deaths has bounds ranging from 257,846 to 722,138. These lower and upper bound values are 57% and 161%, respectively, of the mean estimate (449,813). Correspondingly, the uncertainty of the contribution of each individual anthropogenic source to the PM_2.5_‐associated health impacts ranges from −37.6% to +72.1% in this study. These are likely larger uncertainty ranges than those induced by the exposure estimate, source attribution modeling, or emissions alone as discussed in the previous paragraph.

Apart from uncertainties introduced by technical limitations, our source attribution results might still be limited to some extent when applied in practice. For example, policy makers have a strong interest in designing air quality regulations that are both cost effective and equitable. When regulating polluting industries that are important drivers of economic growth, policy trade‐offs arise which call for careful, quantitative assessments of the economic costs of a specific emission control action, its (monetized) public health benefits, and the geographical distribution of those benefits. This requires an alternative analytical approach that not only identifies where most air pollution‐related health burden comes from but that links policy impacts on the source of emissions to health benefits. To enable this kind of research, future studies will aim to integrate relevant economic and econometric modeling with non‐linear atmospheric chemistry models such as the one we have proposed here. Apart from broadening the scope of the modeling, improving the quality of model inputs would strongly benefit this line of research. First, more detailed mortality data at the sub‐national level, especially for countries covering large areas with dense population, will allow more accurate source attribution and health benefit estimates. Second, advances in the estimation of the concentration‐exposure‐health responses (e.g., Burnett et al., 2018) will reduce biases and large uncertainties in the estimation of health benefits.

## 4 Discussion and Conclusions

In this study, we present a newly developed approach to characterize the sources of PM_2.5_‐related health risks in Europe in 2015 and quantify corresponding changes induced by the anthropogenic emission changes during the first stage of the EU PM_2.5_ objectives from 2005 to 2015, using the CTM GEOS‐Chem and its adjoint. In 2015, the total PM_2.5_‐related premature death is estimated to be 449,813 (257,846–722,138) out of a total population of 598.97 million over the European region considered in this study. Our estimate is slightly lower than that of Lelieveld et al. (2019) since the latter study calculated the PM_2.5_‐associated premature deaths over a larger European region using the Global Exposure Mortality Model, which accounts for a larger range of PM_2.5_ exposure by including new cohort data from China, and providing larger hazard ratio predictions for nearly all concentrations than the GBD estimates (Burnett et al., 2018). IHD and stroke were estimated to be the top two causes of premature death attributable to PM_2.5_ exposure, which is similar to the calculation results reported in recent studies over Europe (Tarín‐Carrasco et al., 2022), China (Zheng et al., 2021), and the US (Kazemiparkouhi et al., 2022).

We find that anthropogenic emissions within Europe contributed 59.0% of the total estimated premature deaths, which is the largest sources of the PM_2.5_‐related health risks in Europe. Due to heterogeneous distributions of precursor species (NO_*x*_, NH_3_, SO_2_, OC, BC, and SOAP), the domestic anthropogenic contributions differed greatly by sector in 2015. Residential and agricultural emissions were the most important contributing sectors, accounting for 23.5%, and 23.0%, respectively, of the total burden of PM_2.5_‐related premature deaths induced by anthropogenic emissions within Europe. Our estimate of residential contributions is likely higher than earlier works owing to the inclusion of SOA. Monthly source apportionment suggests the domestic residential emissions were associated with more premature deaths in winter due to the high emission rates and the high sensitivity for carbonaceous aerosols, while the agricultural emissions led to more premature deaths during February to April when emissions from agricultural crops and waste sources significantly increased before the start of the growing season.

The country‐level source attribution results have multiple policy implications with respect to air quality and public health in Europe. For western and central European countries, anthropogenic emissions from ground transport sectors made up a majority of nationwide contributions to PM_2.5_‐associated premature deaths, while in Mediterranean and Eastern countries, residential emissions were the dominant source of the health risks. However, even for contributions from the same sector, the dominant species can vary by location. The largest diversity is found in industrial and energy sectors, suggesting that the local industrial/energy structures and policies further increase the source complexity of the PM_2.5_‐related health risks. Thus, more detailed source attribution results should be provided at least at the country level so that emissions controls can be better informed and more effective.

Additionally, our calculations suggest that there were redistributions of the anthropogenic contributions within Europe, further increasing the heterogeneity of the pollution related health risks. After excluding the influence of extra‐regional sources, the eastern European countries suffered from more premature deaths than their emissions caused; in contrast, the emissions from some central and western European regions contributed premature deaths exceeding three times the number of deaths that occurred locally. For people living in eastern European countries such as Ukraine, Poland, Romania, they experienced even greater harmful air pollution effects, since they were not only more susceptible to the adverse health effects of the severe local PM_2.5_ pollution but also experience the consequences of emissions from western and central parts of Europe simultaneously, resulting in larger social inequalities with respect to health and other socio‐economic aspects in Europe.

During 2005–2015, emissions controls promoted decreases in the PM_2.5_‐related health risks in nearly all European countries. The anthropogenic emission changes during the 11‐year period resulted in 63,538 (46,092–91,082) fewer PM_2.5_‐related premature deaths in 2015 compared to 2005. Most of the decreases were associated with decreased contributions from ground transport, energy, and industrial sources, making up 53.1%, 29.0%, and 12.8%, respectively, of the total decreases in the premature deaths. This result indicates that the control strategies for these sectors effectively mitigated the detrimental effects of PM_2.5_ pollution on public health in Europe during the first emission control stage. However, there were several sectoral source changes, for example, those in residential, agricultural, waste, shipping, and aviation sources, that had little impact on the estimated decreases and even some that led to an increase in premature deaths during these years. When examining the health impact changes in individual country, we find that countries progress at their own pace in reducing the adverse impacts of air pollution on the public health. Decreases in the contributions from sectoral emissions in some countries can be offset by the increased contributions in others, reducing the benefit of the emission control strategies in some regions. Overall, compared to a focus on local emission reduction policies and actions alone, international cooperation on transboundary air pollution can also be an important part in tacking air pollution and increasing the effectiveness of the EU policies throughout the continent.

## Conflict of Interest

The authors declare no conflicts of interest relevant to this study.

## Supporting information

Supporting Information S1

## Data Availability

The GEOS‐Chem adjoint model used in this study is an open‐access model which is publicly available online (http://wiki.seas.harvard.edu/geos-chem/index.php/GEOS-Chem_Adjoint, accessed on: 11 October 2022). All newly generated data, including the calculated sensitivities of the total PM_2.5_‐related premature deaths to various species emissions in Europe, and the sensitivity results discussed in Section 3.3, is stored in an open repository (Gu et al., 2023).
