# Supplementary material for: Sources of PM2.5‐Associated Health Risks in Europe and Corresponding Emission‐Induced Changes During 2005–2015
Source: Geohealth. 2023 Mar 20;7(3):e2022GH000767. doi: 10.1029/2022GH000767 (PMC10027220; doi:10.1029/2022GH000767)
Supplement: Supplementary file 1 — Supporting Information S1 [file GH2-7-e2022GH000767-s001.docx]

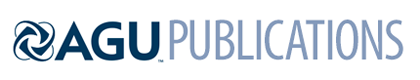


*GeoHealth*

Supporting Information for

**Sources of PM_2.5_-associated Health Risks in Europe and Corresponding Emission-induced Changes during 2005-2015**

Yixuan Gu^1,2^, Daven K. Henze^1^, M. Omar Nawaz^1^, Hansen Cao^3^, and Ulrich J. Wagner^2^

^1^Department of Mechanical Engineering, University of Colorado Boulder, Boulder, CO, 80301, USA

^2^Department of Economics, University of Mannheim, Mannheim, Baden Württemberg, 68161, Germany

^3^Department of Chemistry, University of York, York, YO10 5DD, UK

**Contents of this file**

Text S1 to S7

Figures S1 to S3

Tables S1 to S6

**Introduction**

The supporting information includes detailed introduction of the method of model bias correction and downscaling (Text S1), evaluation of PM_2.5_ in Europe (Text S2), treatment of gases and aerosols in the GEOS-Chem model (Text S3), and calculations of emissions (Text S4 and S5), cost function (Text S6), and emission contributions (Text S7).

Text S1. Model bias correction and downscaling using satellite-derived PM_2.5_

To provide more accurate PM_2.5_ estimates for the cost function calculation, the simulated PM_2.5_ concentrations from the forward model simulation are corrected and downscaled using fine-resolution satellite-derived PM_2.5_ products (van Donkelaar et al., 2021) according to Nawaz et al. (2021). Fine-resolution (0.01°×0.01°) annual mean PM_2.5_ concentrations ($M_{i,j}$) in grid cell ($i$, $j$) are derived as:

$M_{i,j}=M_{I,J}\times(\frac{{SAT}_{I,J}}{M_{I,J}})\times(\frac{{sat}_{i,j}}{{SAT}_{I,J}})$ (1)

where $M_{I,J}$ represents the simulated annual mean PM_2.5_ concentrations from the forward model at the 0.25°×0.3125° resolution in grid cell ($I$, $J$), ${sat}_{i,j}$ is the satellite-based PM_2.5_ estimate at the fine resolution in grid cell ($i$, $j$), and $\mathrm{SAT}_{I,J}$ is the same satellite-based estimate averaged at the coarse model resolution. Note this reduces to directly using the fine-scale satellite-based concentrations for the exposure estimate itself. For each model grid cell, the population-weighted PM_2.5_ concentration ($E_{{PM}_{2.5}}(I,J)$) is then calculated as:

$E_{{PM}_{2.5}}\left( I,J \right)=\frac{\sum_{(i,j)\in D} (M_{i,j}\times{POP}_{i,j})}{\sum_{(i,j)\in D} {POP}_{i,j}}$ (2)

where ${POP}_{i,j}$ is the population in each fine grid cell ($i$, $j$) over the coarse grid cell ($I$, $J$).

Text S2. Evaluation of simulated PM_2.5_ exposure in Europe

To evaluate the simulation performance in estimating PM_2.5_ exposure in the receptor region, simulated surface PM_2.5_ concentrations are compared with a suite of PM_2.5_ observations obtained from 972 surface monitoring sites in Europe. From Figure S1a, the annual mean PM_2.5_ concentrations are underestimated by the forward model in most of the European region, exhibiting a normalized mean bias of -14.7% when compared to the site-averaged annual mean values. Relatively large underestimations are located in Eastern part of the receptor region, which is common across other studies given the uncertainties in the emission inventories in these regions (Im et al., 2015, 2018; Werner et al., 2018; Kryza et al., 2020; Thunis et al., 2021). Previous multi-model studies suggested that although simulated results might exhibit large variability among different models, surface PM_2.5_ concentrations were generally underestimated by 20−60% in Europe using EMEP/CEIP emission inventories (e.g., HTAP_v2.2 in Crippa et al., 2019; EDGARv5.0, EMEP, and CAMS-REF-AP in Thunis et al., 2021), especially in central, eastern European countries and the Mediterranean region. Most of those underestimates were associated with the underestimations of emissions from residential, agricultural, and transport sectors (Im et al., 2015, 2018; Crippa et al., 2019; Kryza et al., 2020). The emission inventory uncertainties and their propagation to PM_2.5_ concentrations can result in large uncertainties in PM_2.5_-related mortality estimates. A simulation from Crippa et al. (2019) suggested that the emission inventory uncertainties could lead to an uncertainty of more than 1 million premature deaths in the estimates of 2.1 million premature deaths per year at the global scale, which was comparable to those induced by uncertainties in the CTM simulations and population exposure functions.

To provide better estimates of PM_2.5_ exposure for the calculations of the related health impacts, improvements from two aspects are applied in this study. As in Eq. (1), the downscaling component, $\frac{{sat}_{i,j}}{{SAT}_{I,J}}$, is used to improve the model’s capability to capture sub-grid variability, providing more spatial information than the model can resolve for surface PM_2.5_ concentrations. The bias correction term, $\frac{{SAT}_{I,J}}{M_{I,J}}$, scales the simulated PM_2.5_ concentrations to the satellite-derived values, which is used to correct biases induced by uncertainties in model simulations (e.g., emissions, schemes…). As Figure S1 shows, the PM_2.5_ underestimation in the central and eastern parts of the receptor region is greatly improved after the bias correction and downscaling are applied. The simulated site-averaged annual mean PM_2.5_ concentration increases from 12.77 µg m^-3^ to 14.08 µg m^-3^, which is closer to the observed level (14.98 µg m^-3^). With over 780 times finer variabilities captured, the correlation coefficient (R) between the simulated and observed values is also significantly improved, with the value of R^2^ increasing from 0.34 to 0.80. The improved PM_2.5_ estimates lead to improved estimates of PM_2.5_ exposure and related premature deaths in Europe.

Text S3. Treatment of gas-phase chemistry and aerosols in the forward model

The forward model corresponds to GEOS-Chem v8-02-01 with updates through model version v9-02, including fully coupled treatment of gas-phase chemistry (Bey et al., 2001; Park et al., 2004) and online aerosol calculations including sulfate ($\mathrm{SO}_{4}^{2-}$), nitrate ($\mathrm{NO}_{3}^{-}$), ammonium ($\mathrm{NH}_{4}^{+}$, Park et al., 2004; Pye et al., 2009), organic carbon (OC) and black carbon (BC, Park et al., 2003), mineral dust (Fairlie et al., 2007) and sea salt (Alexander et al., 2005; Jaeglé et al., 2011). Key features of the inorganic aerosol simulation are similar to those described in Henze et al. (2009). Dry deposition for gas and aerosols is calculated based on the resistance‐in‐series model (Wesely, 1989), and wet deposition follows the scheme of Liu et al. (2001). The hydroscopic growth factors for secondary inorganic aerosols ($\mathrm{SO}_{4}^{2-}$, $\mathrm{NO}_{3}^{-}$, and $\mathrm{NH}_{4}^{+}$,), organic aerosols (hydrophilic OC, SOA), and seal salts are 1.10, 1.05, and 1.86, respectively at 35% RH. The ratio of ambient organic mass (OM) to OC is 2.1 by default.

Text S4. Speciation of NMVOC emissions

NMVOCs include a variety of chemical species which play important roles in atmospheric chemistry, greatly affect SOA formation in the atmosphere (Liao et al., 2007; Henze et al., 2008). In CTMs, these NMVOC species are usually lumped into several groups defined by specific chemical mechanisms according to their structure or reactivity. To map the HTAPv3 mosaic total NMVOCs emissions to lumped, model-ready emissions for the GEOS-Chem, we regrid the emissions to the model resolution, and distribute the total NMVOCs emissions in every grid box to individual species emissions according to the speciation information from the NMVOC EDGARv4.3.2 database (Huang et al., 2017). The monthly emissions of acetone (ACET, C_3_H_6_O), ≥C2 acetaldehyde (ALD2), ≥C4 alkanes (ALK4), ethane (C_2_H_6_), propane (C_3_H_8_), formaldehyde (CH_2_O), methyl ethyl ketone (MEK, C_4_H_8_O), and propene (PRPE, C_3_H_6_) in 2015 are calculated by multiplying the monthly total NMVOC emissions in 2015 by the ratios of each species emissions to total NMVOC emissions according to the monthly gridmaps from EDGARv4.3.2.

Text S5. Calculations of anthropogenic emissions of SOA precursors

For anthropogenic emissions of SOA precursors (SOAP), following Nault et al. (2021), the ratios of monthly SOAP emissions to CO emissions are firstly calculated using emissions of CO, benzene, toluene, and xylene from EDGARv4.3.2. The monthly SOAP emissions in each model box are then determined by multiplying the ratio with monthly CO emissions in 2015.

Text S6. Cost function

The cost function in this study this is defined as the total number of PM_2.5_-related premature deaths in all the European countries listed in the Global Health Data Exchange (GHDx, <https://ghdx.healthdata.org/>, accessed on: 11 October 2022) over the targeted receptor region $D$ (shaded areas in Fig.1) in 2015:

$J_{{PM}_{2.5}}=\sum_{L} \sum_{A} \sum_{k\in D} \sum_{(I,J)\in k} ({POP}_{I,J,A}\times{MOR}_{I,J,A,L}\times{AF}_{I,J,A,L})$ (3)

where $I$ and $J$ are 0.25°×0.3125° model grid indices in country $k$ belonging to the receptor region $D$, and $L$ is the health outcome (COPD, IHD, LRO, LC, T2D, and stroke) that can be affected by PM_2.5_ pollution suggested by the Global Burden of Disease Study (GBD) 2019 (Murray et al., 2020). ${POP}_{I,J,A}$ is the population of age group $A$ in grid cell ($I$, $J$), which is calculated by multiplying the total population by corresponding proportion of the population in age group $A$ for the base year of 2015. The gridded total population is obtained from fine resolution (~1 km) population estimate of Center for International Earth Science Information Network (CIESIN, 2018), and the ratio of each age group to the total population is obtained from the GBD Results Tool (<https://vizhub.healthdata.org/gbd-results/>, accessed on: 11 October 2022). ${MOR}_{I,J,A,L}$ is the age-specific baseline mortality of cause $L$ in country $k$, which is also retrieved from the GBD Results Tool. ${AF}_{I,J,A,L}$ is the age-specific attributable fraction of deaths from cause $L$ in grid cell ($I$, $J$), which is calculated as:

${AF}_{I,J,A,L}=\frac{{RR}_{I,J,A,L}-1}{{RR}_{I,J,A,L}}$ (4)

Here, ${RR}_{I,J,A,L}$ is the relative risk determined by the updated PM_2.5_ exposure in grid cell ($I$, $J$) according to the integrated exposure response relationships in GBD 2019 study (Murray et al., 2020). The age-specific relative risks for COPD, IHD, LRO, LC, T2D, and stroke are calculated from look-up tables which relate exposure levels to age-specific relative risks for these health outcomes, and are linearly interpolated between adjacent exposure levels.

Text S7. Calculation of emission contributions

To better understand the regional sources of PM_2.5_-associated health risks in the target region, we consider the gradients ($\lambda_{E}=\frac{\partial J_{{PM}_{2.5}}}{\partial E}$) at the resolution of the HTAP v3 emissions (0.1°×0.1°) by assuming the sensitivities are constant over each model grid cell (0.25°×0.3125°). The unique contribution of emissions from a specific species $p$, sector $s$, month $t$, and fine grid cell ($i$, $j$) on the cost function is then determined as:

$dJ_{i,j,p,s,t}=\lambda_{i,j,p,t}\times E_{i,j,p,s,t}$ (5)

where $p$ is any of the six PM_2.5_ precursors (SO_2_, NO_x_, NH_3_, OC, BC, SOAP), $s$ can be any detailed sector in the HTAPv3 emission inventory listed in Table S1, $E_{i,j,p,s,t}$ represents the HTAPv3 emission for the same $p$ and sector $s$ during month $t$ in grid cell ($i$, $j$) as the sensitivity $\lambda_{i,j,p,t}$.


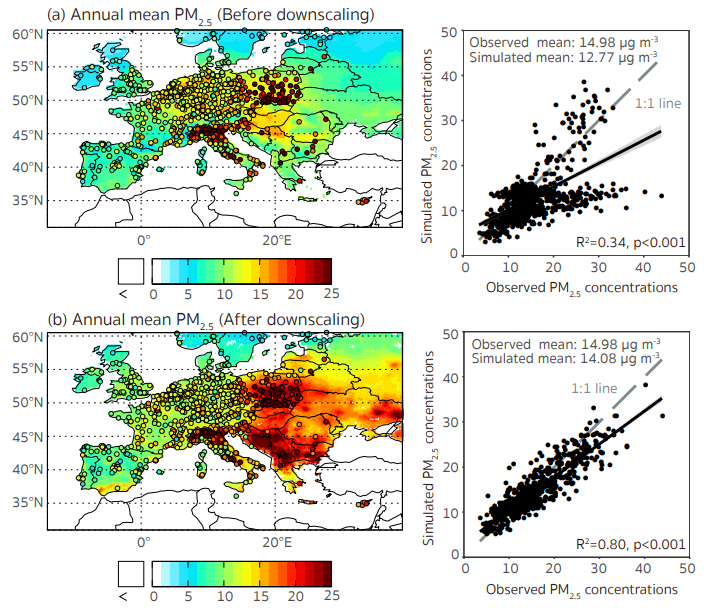


Figure S1. Evaluation of simulated annual mean PM_2.5_ concentrations (unit: µg m^-3^) (a) before and (b) after the satellite downscaling. In the left column are the simulated annual mean surface PM_2.5_ concentrations with observed values (dotted) mapped. In the right column are the scatter plots which compare the simulated values with in-situ observed values included in the receptor region.


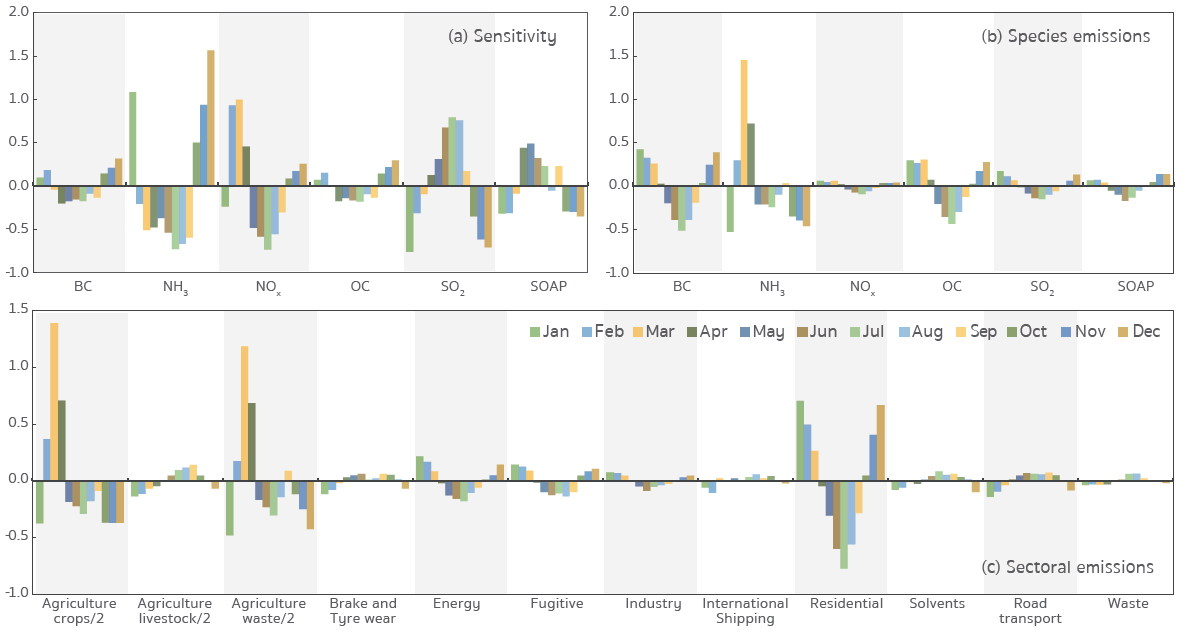


Figure S2. The normalized monthly variations of (a) mean species sensitivities, and total emissions from each individual (b) species and (c) sector over the nested European domain. The normalized change is calculated as $\boldsymbol{NMC=}\frac{\left( \boldsymbol{V}_{\boldsymbol{m}}\boldsymbol{-}\overline{\boldsymbol{V}} \right)}{\overline{\boldsymbol{V}}}\boldsymbol{\times100\%}$, where $\boldsymbol{V}_{\boldsymbol{m}}$ is the value of mean sensitivity or emission in month $\boldsymbol{m}$, and $\overline{\boldsymbol{V}}$ is the annual mean value. Only sectoral emissions with normalized changes larger than 5% are included in (c). The normalized changes in emissions from agriculture crops, agriculture livestock, and agriculture waste sectors are two times the values displayed in the figure.


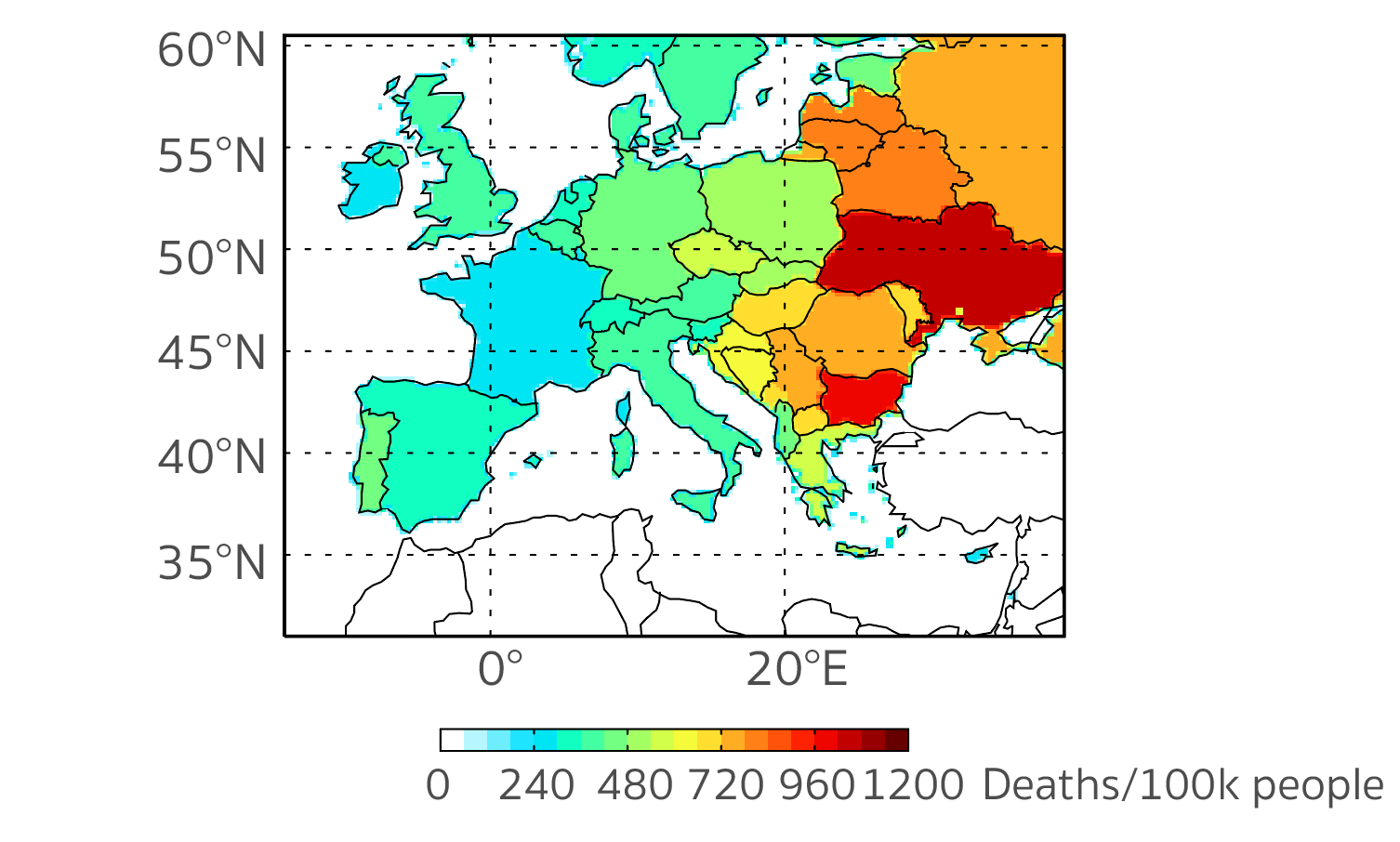


Figure S3. The country-specific total mortalities of the PM_2.5_-related health outcomes (IHD, COPD, LRI, LC, T2D, and STROKE) in 2015 according the GBD study in 2019.Table S1. Sector definitions for the HTAPv3 mosaic emission inventory used in this study.

| Main sectors | Detailed sectors |
| --- | --- |
| Shipping | International shipping |
|  | Domestic shipping |
| Aviation | International aviation |
|  | Domestic aviation |
| Energy | Energy |
| Industry | Industry |
|  | Fugitive |
|  | Solvents |
| Ground transport | Road transport |
|  | Brake and Tyre wear |
|  | Other ground transport |
| Residential | Residential |
| Waste | Waste |
| Agriculture | Agricultural waste burning |
|  | Agriculture-livestock |
|  | Agriculture-crops |

Table S2. Estimates of PM_2.5_-related premature deaths in Europe from this study and previous model studies.

| PM_2.5_-related premature death (per annum) | Year | Exposure Resolution | Exposure–response model | Include the FSU (former Soviet Union) or not | Reference |
| --- | --- | --- | --- | --- | --- |
| 546,000 | 1997-2003 mean | 0.4°×0.4° | Linear (Pope et al. 2002;  Jerrett et al., 2005) | No | Andersson et al., 2009 |
| 680,000 | 2000 | 50km×50km | Linear (Pope et al. 2002) | Parts of | Brandt et al., 2013 |
| 450,000 | 2020 |  |  |  |  |
| 374,000 | 2010 | 1.1°×1.1° | GBD 2010 study (Lim et al., 2012) | Yes | Lelieveld et al., 2015 |
| 403,000 | 2012 | Observation-based | Linear (Henschel, et al., 2013) | No | EEA, 2015 |
| 507,588 | 2018 |  |  |  | EEA,2020 |
| 295,519−420,575 | 2000 | 50km×50km | Linear (Pope et al. 2002) | No | Geels et al., 2015 |
| 236,000 (anthropogenic) | 2005 | 0.67°×0.5° | GBD 2010 study (Burnett et al., 2014) | No | Silva, Adelman et al., 2016 |
| 180,000−550,000 | 2010 | 0.25°×0.25° | Linear (Pope et al. 2002) | No | Im et al., 2018 |
| 645,000−934,000 | 2015 | 1.1°×1.1° | GEMM (Burnett et al. 2018) | No | Lelieveld et al., 2019 |
| 960,419 | 1990 | 0.25°×0.4° | Linear (Henschel, et al., 2013) | No | Ciarelli et al., 2019 |
| 498,168 | 2010 |  |  |  |  |
| 444,535 | 2015 |  |  |  |  |
| 8,626,000 | 2012 | 0.5°×0.67° | Linear (Vodonos et al., 2018) | Yes | Vohra et al., 2021 |
| 725,000−1,056,000 | 2010 | 0.11°×0.11° | GEMM (Burnett et al.2018) | Parts of | Tarín-Carrasco et al., 2022 |
| **261,383−650,710** | **2015** | **0.01°×0.01°** | **GBD 2019 study (Murray et al., 2020)** | **Parts of** | **This study** |

Table S3. PM_2.5_-related premature deaths contributed by the anthropogenic emissions from each individual species and sector within Europe in 2015.

| Main sectors | Detail sectors | NO_x_ | NH_3_ | SO_2_ | OC | BC | SOAP | **Total** |
| --- | --- | --- | --- | --- | --- | --- | --- | --- |
| Shipping | International shipping | 6,661 | 2 | 3,154 | 428 | 402 | 175 | **10,822** |
|  | Domestic shipping | 964 | 0 | 195 | 97 | 71 | 72 | **1,399** |
| Aviation | International aviation | 6,102 | 294 | 302 | 74 | 71 | 158 | **7,001** |
|  | Domestic aviation | 295 | 27 | 21 | 7 | 6 | 31 | **387** |
| Energy | Energy | 11,557 | 355 | 14,933 | 575 | 676 | 1,127 | **29,223** |
| Industry | Industry | 9,263 | 2,265 | 5,936 | 5,680 | 2,042 | 7,662 | **32,848** |
|  | Fugitive | 261 | 665 | 287 | 239 | 803 | 330 | **2,585** |
|  | Solvents | 32 | 728 | 5 | - | - | 116 | **881** |
| Ground transport | Road transport | 26,191 | 1,808 | 70 | 1,640 | 1,830 | 8,211 | **39,750** |
|  | Brake and Tire wear | - | - | - | 807 | 125 | - | **932** |
|  | Other ground transport | 6,123 | 32 | 89 | 1,194 | 894 | 2,331 | **10,663** |
| Residential | Residential | 6,342 | 3,270 | 1,705 | 26,257 | 13,183 | 11,625 | **62,382** |
| Waste | Waste | 354 | 1,820 | 17 | 1,853 | 613 | 653 | **5,310** |
| Agriculture | Agricultural waste burning | 307 | 217 | 16 | 1,629 | 285 | 633 | **3,087** |
|  | Agriculture-livestock | 712 | 38,821 | - | 3,226 | - | - | **42,759** |
|  | Agriculture-crops | 3,391 | 11,127 | - | 780 | - | 1 | **15,299** |
| **Total** | | **78,555** | **61,431** | **26,730** | **44,486** | **21,001** | **33,125** | **265,328** |

Table S4. Contributions of sectoral emissions from each individual country/region (with total nationwide contributions larger than 10 premature deaths) to the PM_2.5_-related premature deaths in Europe in 2015.

| Country name | AGRC | AGRL | AGRW | BRAY | DOAV | DOSH | ENER | FUGI | INAV | INDU | INSH | OGTR | RESI | SOLV | RTRA | WAST | TOTAL |
| --- | --- | --- | --- | --- | --- | --- | --- | --- | --- | --- | --- | --- | --- | --- | --- | --- | --- |
| Ukraine | 1,213 | 6,208 | 2,050 | 30 | 2 | 96 | 6,673 | 262 | 237 | 6,769 | 40 | 1,742 | 5,156 | 0 | 2,120 | 1,983 | 34,581 |
| Germany | 2,095 | 5,741 | 4 | 144 | 74 | 266 | 3,078 | 120 | 1,131 | 5,031 | 287 | 1,765 | 2,830 | 120 | 7,746 | 151 | 30,581 |
| Poland | 1,321 | 3,315 | 12 | 47 | 2 | 1 | 3,870 | 421 | 229 | 2,075 | 52 | 577 | 5,478 | 14 | 2,752 | 117 | 20,282 |
| Italy | 884 | 3,342 | 50 | 89 | 40 | 2 | 288 | 195 | 622 | 1,279 | 147 | 570 | 7,922 | 21 | 4,242 | 411 | 20,105 |
| Russia* | 1,746 | 1,706 | 594 | 52 | 47 | 17 | 2,947 | 414 | 165 | 3,318 | 81 | 304 | 4,885 | 0 | 2,286 | 143 | 18,705 |
| France | 1,440 | 2,972 | 3 | 120 | 49 | 42 | 301 | 16 | 684 | 1,706 | 71 | 983 | 3,660 | 18 | 4,000 | 152 | 16,217 |
| Romania | 883 | 2,001 | 99 | 21 | 5 | 35 | 1,340 | 49 | 233 | 1,073 | 46 | 366 | 6,052 | 5 | 1,497 | 332 | 14,036 |
| United Kingdom | 989 | 1,549 | 1 | 124 | 33 | 1 | 885 | 31 | 518 | 1,765 | 100 | 949 | 2,334 | 405 | 2,233 | 209 | 12,125 |
| Spain | 588 | 1,716 | 17 | 47 | 39 | 3 | 702 | 34 | 245 | 1,371 | 84 | 321 | 2,418 | 14 | 1,353 | 37 | 8,989 |
| Serbia | 174 | 1,398 | 23 | 5 | 0 | 14 | 2,307 | 138 | 107 | 338 | 0 | 198 | 1,904 | 0 | 403 | 355 | 7,365 |
| Hungary | 380 | 1,332 | 8 | 15 | 0 | 19 | 276 | 17 | 171 | 282 | 0 | 209 | 3,494 | 6 | 939 | 41 | 7,190 |
| Czechia | 415 | 1,085 | 1 | 17 | 5 | 0 | 836 | 58 | 163 | 697 | 0 | 252 | 2,789 | 7 | 842 | 14 | 7,182 |
| Belarus | 153 | 1,620 | 32 | 6 | 3 | 11 | 501 | 9 | 75 | 439 | 0 | 500 | 707 | 0 | 626 | 252 | 4,933 |
| Bulgaria | 255 | 779 | 77 | 12 | 1 | 47 | 589 | 41 | 78 | 439 | 10 | 67 | 1,633 | 5 | 558 | 106 | 4,698 |
| Austria | 201 | 747 | 0 | 14 | 4 | 13 | 145 | 52 | 263 | 600 | 0 | 247 | 757 | 4 | 1,217 | 64 | 4,328 |
| Netherlands | 328 | 1,007 | 0 | 20 | 1 | 194 | 131 | 84 | 162 | 289 | 309 | 251 | 273 | 189 | 883 | 16 | 4,137 |
| Belgium | 203 | 812 | 0 | 24 | 0 | 42 | 84 | 6 | 141 | 698 | 183 | 148 | 789 | 7 | 813 | 5 | 3,958 |
| Bosnia and Herzegovina | 27 | 548 | 1 | 4 | 1 | 0 | 1,051 | 157 | 63 | 209 | 0 | 8 | 1,020 | 0 | 312 | 94 | 3,494 |
| Slovakia | 186 | 466 | 0 | 7 | 0 | 3 | 376 | 108 | 54 | 519 | 0 | 74 | 1,059 | 3 | 415 | 29 | 3,298 |
| Croatia | 137 | 340 | 0 | 5 | 0 | 2 | 87 | 10 | 96 | 180 | 15 | 83 | 1,438 | 2 | 289 | 19 | 2,703 |
| Switzerland | 129 | 496 | 0 | 25 | 9 | 1 | 35 | 1 | 239 | 159 | 0 | 221 | 343 | 26 | 758 | 23 | 2,466 |
| Greece | 238 | 291 | 6 | 12 | 1 | 4 | 245 | 119 | 41 | 223 | 22 | 52 | 635 | 8 | 343 | 0 | 2,239 |
| Portugal | 59 | 174 | 3 | 8 | 1 | 2 | 39 | 6 | 31 | 326 | 16 | 25 | 560 | 3 | 318 | 14 | 1,585 |
| North Macedonia | 15 | 268 | 3 | 2 | 0 | 0 | 291 | 27 | 21 | 162 | 0 | 5 | 511 | 0 | 98 | 82 | 1,485 |
| Republic of Moldova | 42 | 417 | 29 | 1 | 0 | 1 | 99 | 1 | 24 | 23 | 0 | 28 | 316 | 0 | 114 | 303 | 1,397 |
| Kosovo | 20 | 177 | 2 | 3 | 0 | 0 | 358 | 6 | 19 | 21 | 0 | 30 | 438 | 0 | 194 | 114 | 1,382 |
| Slovenia | 55 | 211 | 2 | 4 | 0 | 0 | 68 | 2 | 53 | 91 | 6 | 65 | 496 | 1 | 228 | 1 | 1,282 |
| Denmark | 174 | 294 | 0 | 5 | 1 | 3 | 54 | 2 | 25 | 44 | 18 | 95 | 247 | 3 | 217 | 10 | 1,191 |
| Lithuania | 199 | 167 | 2 | 1 | 1 | 2 | 74 | 33 | 31 | 95 | 2 | 37 | 155 | 1 | 214 | 10 | 1,024 |
| Sweden* | 111 | 178 | 0 | 27 | 2 | 2 | 41 | 2 | 46 | 70 | 15 | 120 | 122 | 0 | 151 | 22 | 909 |
| Ireland | 149 | 176 | 4 | 4 | 0 | 1 | 62 | 2 | 46 | 68 | 18 | 22 | 149 | 0 | 206 | 0 | 907 |
| Latvia | 68 | 108 | 10 | 2 | 0 | 0 | 50 | 10 | 29 | 72 | 18 | 58 | 353 | 2 | 76 | 6 | 863 |
| Albania | 17 | 260 | 2 | 3 | 0 | 0 | 7 | 2 | 18 | 46 | 2 | 14 | 142 | 0 | 182 | 54 | 748 |
| Norway* | 43 | 41 | 0 | 3 | 2 | 19 | 1 | 1 | 19 | 55 | 57 | 55 | 130 | 0 | 56 | 5 | 487 |
| Estonia | 46 | 61 | 5 | 1 | 0 | 0 | 41 | 4 | 8 | 44 | 8 | 20 | 61 | 0 | 30 | 1 | 331 |
| Montenegro | 10 | 38 | 10 | 0 | 0 | 0 | 62 | 1 | 15 | 2 | 5 | 4 | 97 | 0 | 41 | 21 | 307 |
| Luxembourg | 14 | 56 | 3 | 2 | 0 | 0 | 7 | 0 | 14 | 36 | 0 | 17 | 42 | 0 | 69 | 0 | 259 |
| Finland* | 17 | 18 | 0 | 8 | 0 | 0 | 16 | 10 | 12 | 22 | 3 | 18 | 46 | 3 | 27 | 8 | 208 |
| Cyprus | 2 | 9 | 0 | 0 | 0 | 0 | 3 | 0 | 1 | 1 | 0 | 0 | 0 | 0 | 3 | 0 | 20 |
| Malta | 1 | 1 | 0 | 0 | 0 | 0 | 2 | 0 | 1 | 0 | 3 | 0 | 1 | 0 | 2 | 0 | 10 |

*Only part of the country territory included in the nested model domain

AGRC=Agriculture crops; AGRL=Agriculture livestock; AGRW=Agriculture waste; BRAY=Brake and Tyre; DOAV=Domestic aviation; DOSH=Domestic shipping; ENER=Energy; FUGI=Fugitive; INAV=International aviation; INDU=Industry; INSH=International shipping; OGTR=Other ground transport; RESI=Residential; SOLV=Solvent; RTRA=Road transport; WAST=Waste

Table S5. Contributions of species emissions from each individual country/region (with total nationwide contributions larger than 10 premature deaths) to the PM_2.5_-related premature deaths in Europe in 2015.

| Country name | BC | NH_3_ | NO_x_ | OC | SO_2_ | SOAP | Total |
| --- | --- | --- | --- | --- | --- | --- | --- |
| Ukraine | 3,518 | 7,294 | 5,656 | 7,547 | 6,585 | 3,980 | 34,581 |
| Germany | 860 | 7,968 | 11,724 | 1,896 | 1,459 | 6,674 | 30,581 |
| Poland | 1,963 | 5,024 | 5,942 | 2,261 | 3,149 | 1,943 | 20,282 |
| Italy | 1,383 | 4,325 | 6,224 | 4,450 | 350 | 3,372 | 20,105 |
| Russia* | 1,146 | 4,888 | 3,102 | 5,824 | 1,618 | 2,126 | 18,705 |
| France | 1,059 | 4,296 | 6,619 | 2,041 | 495 | 1,707 | 16,217 |
| Romania | 2,029 | 3,081 | 3,425 | 3,608 | 1,101 | 792 | 14,036 |
| United Kingdom | 990 | 2,969 | 4,120 | 2,223 | 566 | 1,258 | 12,125 |
| Spain | 675 | 2,237 | 2,521 | 1,452 | 628 | 1,476 | 8,989 |
| Serbia | 841 | 1,625 | 1,213 | 1,426 | 1,651 | 608 | 7,365 |
| Hungary | 932 | 1,815 | 1,692 | 1,624 | 156 | 971 | 7,190 |
| Czechia | 565 | 1,699 | 1,913 | 960 | 618 | 1,426 | 7,182 |
| Belarus | 310 | 1,533 | 1,600 | 745 | 380 | 366 | 4,933 |
| Bulgaria | 634 | 1,324 | 935 | 978 | 632 | 195 | 4,698 |
| Austria | 178 | 1,019 | 2,089 | 312 | 92 | 637 | 4,328 |
| Netherlands | 116 | 1,337 | 1,823 | 355 | 159 | 347 | 4,137 |
| Belgium | 283 | 978 | 1,439 | 561 | 170 | 526 | 3,958 |
| Bosnia and Herzegovina | 385 | 575 | 589 | 620 | 926 | 399 | 3,494 |
| Slovakia | 275 | 758 | 848 | 451 | 405 | 561 | 3,298 |
| Croatia | 398 | 569 | 625 | 776 | 73 | 262 | 2,703 |
| Switzerland | 118 | 636 | 1,174 | 191 | 26 | 321 | 2,466 |
| Greece | 264 | 680 | 419 | 425 | 207 | 245 | 2,239 |
| Portugal | 223 | 302 | 384 | 413 | 78 | 186 | 1,585 |
| North Macedonia | 185 | 289 | 217 | 289 | 301 | 204 | 1,485 |
| Republic of Moldova | 164 | 384 | 324 | 423 | 18 | 83 | 1,397 |
| Kosovo | 184 | 254 | 252 | 286 | 255 | 152 | 1,382 |
| Slovenia | 130 | 312 | 458 | 276 | 33 | 74 | 1,282 |
| Denmark | 80 | 433 | 393 | 159 | 28 | 98 | 1,191 |
| Lithuania | 46 | 371 | 356 | 91 | 88 | 72 | 1,024 |
| Sweden* | 38 | 315 | 282 | 111 | 25 | 139 | 909 |
| Ireland | 62 | 256 | 415 | 89 | 45 | 39 | 907 |
| Latvia | 136 | 228 | 199 | 239 | 20 | 40 | 863 |
| Albania | 73 | 248 | 215 | 150 | 23 | 40 | 748 |
| Norway* | 55 | 94 | 156 | 98 | 19 | 65 | 487 |
| Estonia | 25 | 117 | 87 | 36 | 45 | 20 | 331 |
| Montenegro | 36 | 53 | 72 | 67 | 47 | 32 | 307 |
| Luxembourg | 9 | 71 | 120 | 20 | 6 | 33 | 259 |
| Finland* | 17 | 57 | 55 | 38 | 23 | 18 | 208 |
| Cyprus | 1 | 10 | 3 | 3 | 3 | 1 | 20 |
| Malta | 0 | 2 | 4 | 1 | 3 | 1 | 10 |

*Only part of the country territory included in the nested model domain.

Table S6. The contribution ratio (CR) in each European country (region). Countries are listed in a descending order according to the value of anthropogenic emission contributed premature deaths divided by the estimated premature deaths occurred locally (CR1).

| Country name | CR1 (relative to total estimated PM_2.5_-related premature deaths) | CR2 (relative to total deaths contributed by sources within Europe) |
| --- | --- | --- |
| Luxembourg | 1.39 | 1.93 |
| Slovenia | 1.02 | 1.16 |
| Austria | 0.99 | 1.22 |
| Estonia | 0.98 | 1.36 |
| Switzerland | 0.84 | 1.09 |
| Czechia | 0.83 | 1.02 |
| Ireland | 0.79 | 1.32 |
| France | 0.73 | 0.99 |
| Belgium | 0.72 | 1.08 |
| Germany | 0.71 | 0.97 |
| Norway* | 0.71 | 1.15 |
| Slovakia | 0.69 | 0.81 |
| Croatia | 0.64 | 0.73 |
| Serbia | 0.63 | 0.73 |
| Bosnia and Herzegovina | 0.63 | 0.74 |
| Hungary | 0.62 | 0.71 |
| Denmark | 0.60 | 0.83 |
| Netherlands | 0.60 | 0.87 |
| Poland | 0.58 | 0.71 |
| Romania | 0.57 | 0.68 |
| Italy | 0.54 | 0.63 |
| Spain | 0.52 | 0.82 |
| Ukraine | 0.48 | 0.66 |
| United Kingdom | 0.48 | 0.77 |
| Belarus | 0.45 | 0.60 |
| Portugal | 0.43 | 0.69 |
| Sweden* | 0.43 | 0.64 |
| Finland* | 0.43 | 0.79 |
| Kosovo | 0.43 | 0.51 |
| Montenegro | 0.41 | 0.54 |
| North Macedonia | 0.41 | 0.50 |
| Russia* | 0.40 | 0.72 |
| Latvia | 0.37 | 0.48 |
| Albania | 0.35 | 0.45 |
| Republic of Moldova | 0.35 | 0.44 |
| Lithuania | 0.34 | 0.44 |
| Bulgaria | 0.34 | 0.42 |
| Greece | 0.30 | 0.42 |
| Andorra | 0.14 | 0.23 |
| Cyprus | 0.06 | 0.12 |
| Isle of Man | 0.06 | 0.10 |
| Jersey | 0.05 | 0.08 |
| Northern Cyprus | 0.02 | 0.04 |

*Only part of the country territory included in the nested model domain.
